# Diverse panicle architecture results from various combinations of *Prl5/GA20ox4 and Pbl6/APO1* alleles

**DOI:** 10.1038/s42003-020-1036-8

**Published:** 2020-06-11

**Authors:** Ayumi Agata, Koki Ando, Sadayuki Ota, Mikiko Kojima, Yumiko Takebayashi, Sayaka Takehara, Kazuyuki Doi, Miyako Ueguchi-Tanaka, Takamasa Suzuki, Hitoshi Sakakibara, Makoto Matsuoka, Motoyuki Ashikari, Yoshiaki Inukai, Hidemi Kitano, Tokunori Hobo

**Affiliations:** 1 Graduate School of Bioagricultural Sciences, Nagoya University, Nagoya, Aichi 464-8601 Japan; 2 RIKEN Center for Sustainable Resource Science, Kanagawa, 230-0045 Japan; 3 Bioscience and Biotechnology Center, Nagoya University, Nagoya, Aichi 464-8601 Japan; 4 College of Bioscience and Biotechnology, Chubu University, Kasugai, Aichi 478-8501 Japan; 5 International Center for Research and Education in Agriculture, Nagoya University, Nagoya, Aichi 464-8601 Japan

**Keywords:** Plant morphogenesis, Gibberellins, Plant breeding, Plant morphogenesis, Gibberellins

## Abstract

Panicle architecture directly affects crop productivity and is a key target of high-yield rice breeding. Panicle length strongly affects panicle architecture, but the underlying regulatory mechanisms are largely unknown. Here, we show that two quantitative trait loci (QTLs), *PANICLE RACHIS LENGTH5* (*Prl5*) and *PRIMARY BRANCH LENGTH6* (*Pbl6*), independently regulate panicle length in rice. *Prl5* encodes a gibberellin biosynthesis enzyme, OsGA20ox4. The expression of *Prl5* was higher in young panicles resulting in panicle rachis elongation. *Pbl6* is identical to *ABERRANT PANICLE ORGANIZATION 1* (*APO1*), encoding an F-box-containing protein. We found a novel function that higher expression of *Pbl6* is responsible for primary branch elongation. RNA-seq analysis revealed that these two genes independently regulate panicle length at the level of gene expression. QTL pyramiding of both genes increased panicle length and productivity. By combining these two genes in various combinations, we designed numerous panicle architecture without trade-off relationship.

## Abstract (web summary)

Ayumi Agata et al. study the molecular mechanisms regulating panicle length which directly affects crop yield. They identify QTLs *Prl5* and *Pbl6* that independently regulate panicle length in rice at the gene expression level. By designing different allelic combinations, they generate desired panicle architecture and confirm their positive effect on yield in the field.

## Introduction

**Fig. 1:**
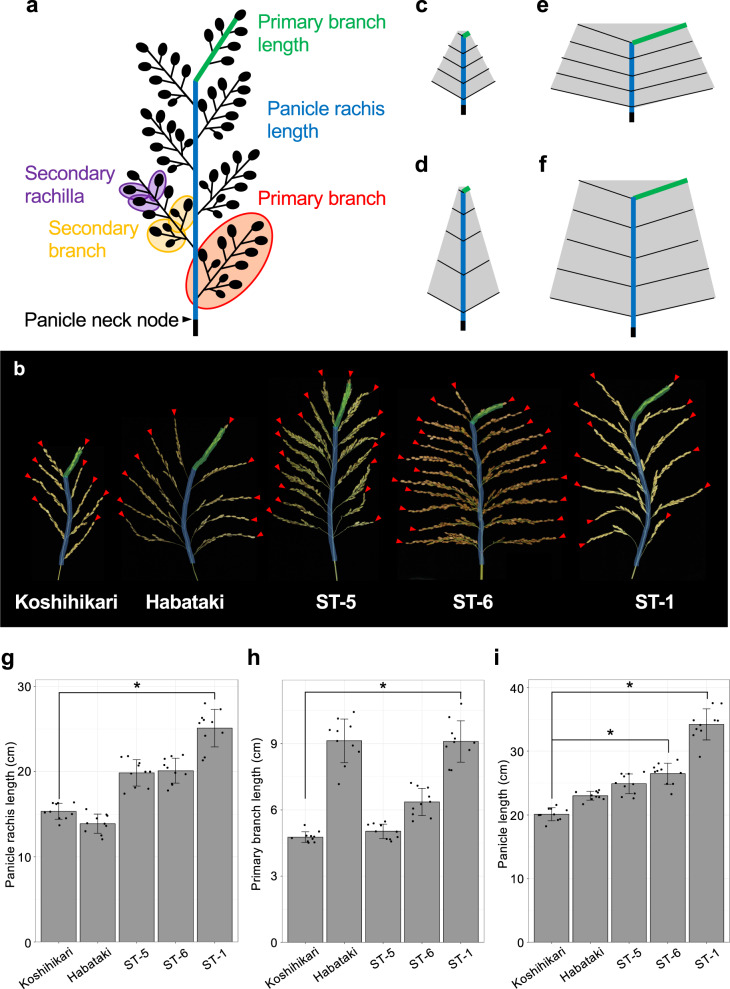
Characterization of rice panicle architecture. **a** Schematic diagram of a rice inflorescence. **b** Panicle phenotypes of five natural rice varieties. Green bars indicate the primary branch tips. Blue bars indicate panicle rachises. Red arrowheads indicate primary branches. Scale bar: 5 cm. **c**–**f** Diagram of four types of panicle architecture with different panicle lengths. Panel **d** has a longer panicle rachis than **c**. Panel **e** has longer primary branches than **c**. Panel **f** has a longer panicle rachis and primary branches than **c**. **g**–**i** Comparison of panicle traits. **g** Panicle rachis length. **h** Primary branch length. **i** Panicle length. Error bars represent means ± SD (*n* = 10 plants). *Significant at the 5% level (Tukey’s significant difference test).

Rice panicle architecture has long been a key target of high-yield rice breeding. Panicle architecture involves several organs, including the panicle rachis, primary branches, and secondary branches (Fig. 1a)^1^. Primary branches develop from the panicle rachis, and each primary branch gives rise to secondary branches. Panicle branching patterns that are mainly regulated by the number of primary and secondary branches directly determine total grain number. However, improving grain productivity by increasing panicle branching alone is limited, as panicle length and branch length remain constant. Thus, both panicle length and the length of each branch are also important factors determining grain productivity. Natural rice varieties show various panicle phenotypes due to gradual changes in these organs (Fig. 1b). Some varieties have increased numbers of primary branches, and others have increased numbers of secondary branches on each primary branch. Some varieties have longer panicle rachises and others have longer primary branches. Some varieties tend to keep primary branch length longer from lower to upper axis. If the panicle rachis length is constant, the number of primary branches that develop from the panicle rachis is limited. Primary branch length also limits the promotion of secondary branch initiation. Many high-yielding rice cultivars tend to have longer primary branches and produce more secondary branches than standard varieties. Therefore, panicle rachis length and primary branch length influence total grain number and rice productivity as well as branching number. Panicle length strongly affects panicle architecture. Panicle length involves two components: panicle rachis length and the length of the top primary branch. For instance, if only panicle rachis length increases, the plants have a panicle architecture like that shown in Fig. 1d compared to Fig. 1c. If only primary branch length increases, the plants have a panicle architecture like that shown in Fig. 1e. If both panicle rachis and primary branch length increase, the plants have a panicle architecture like that shown in Fig. 1f. Therefore, even if the sizes of only two organs change, the plants show various panicle architectures. Thus, panicle length is a key factor determining the diversity of panicle architecture in rice.

Several genes related to panicle morphogenesis have been cloned, some of which were already shown to be agronomically important. *Grain Number 1a* (*GN1a*)^2^, *WEALTHY FARMER’S PANICLE* (*WFP*)^3^, *ABERRANT PANICLE ORGANIZATION1* (*APO1*)^4,5^, and *FRIZZY PANICLE* (*FZP*)^6–8^ significantly increase branch number and total grain number. However, few genes related to panicle length have thus far been cloned. A natural allele of *DENSE AND ERECT PANICLE1* (*DEP1*) regulates panicle length^9^. Specifically, *DEP1* improves yield potential by pleiotropically reducing panicle internode length while increasing grain number. Identifying novel genes related to panicle length would help reveal the fundamental mechanisms that determine various types of variety-specific panicle architecture. Furthermore, these analyses further promote the rational design of ideal panicle architecture for producing elite crop varieties with high yield^10,11^.

In this study, we performed QTL analysis and identify two QTLs for panicle length from ST-1 (from the Stocked rice collections of Togo field and Nagoya University-1). ST-1 possesses longer panicles than Japanese rice cultivar, Koshihikari. The QTLs were mapped to *PANICLE RACHIS LENGTH5* (*Prl5*) and *PRIMARY BRANCH LENGTH6* (*Pbl6*). *Pbl6* contains *APO1*, a gene previously reported to regulate panicle development. *Prl5* encodes Gibberellin 20 oxidase-4. By combining *Pbl6* and *Prl5*, we produced NILs with longer panicles and increased productivity.

## Results

### Two QTLs regulate panicle length

**Fig. 2:**
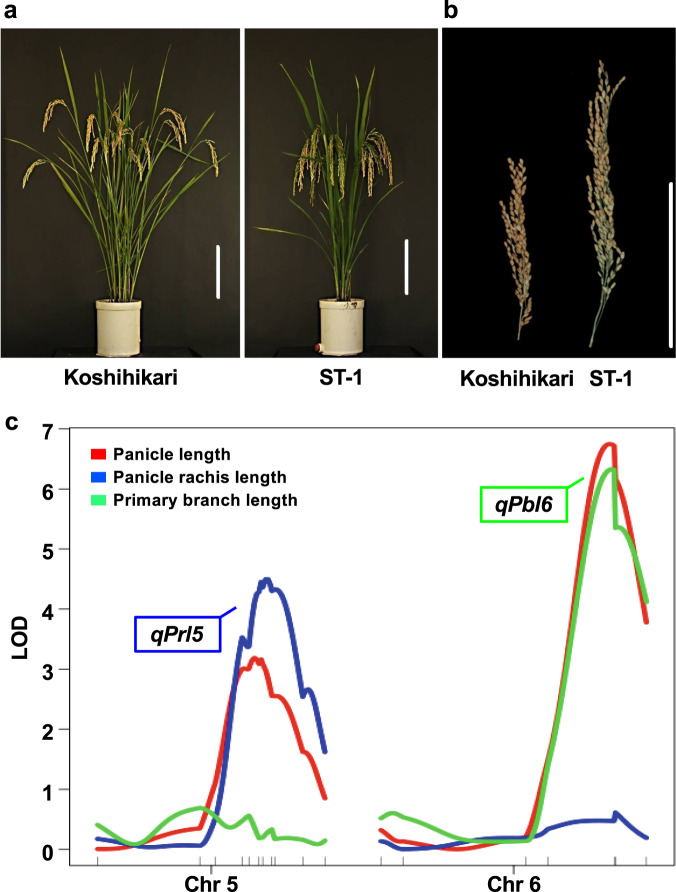
QTL analysis. **a** Gross morphologies of Koshihikari and ST-1. Scale bar: 20 cm. **b** Panicle morphologies of Koshihikari and ST-1. Scale bar: 20 cm. **c** QTL locations for panicle length, panicle rachis length, and primary branch length. Red bars indicate results for panicle length. Blue bars indicate results for panicle rachis length. Green bars indicate results for primary branch length, respectively. QTLs for panicle length and panicle rachis length were detected at the same position on chromosome 5. QTLs for panicle length and primary branch length were detected at the same position on chromosome 6.

Natural rice varieties show wide phenotypic variation in panicle length. We selected five natural rice plants characterized by panicle length from the Stocked rice collections of the Togo field and Nagoya University (Fig. 1b). As panicle length is determined by primary branch length and panicle rachis length (Fig. 1a), we measured each organ (Fig. 1g–i). Koshihikari has short panicle like Fig. 1c. Habataki has short panicle rachis but quite long primary branches like Fig. 1e. ST-6 and ST-5 show middle phenotypes between Fig. 1d and f. ST-1 have both longer panicle rachis and primary branches like Fig. 1f. There is a distinct difference between Koshihikari and ST-1 in panicle length and its components (Fig. 1g–i). The main panicles of Koshihikari and ST-1, which has short culms but extremely long panicles (Fig. 2a, b), are ~20 and 35 cm long, respectively (Fig. 1i). Thus, to investigate genes that regulate panicle length, we selected these two rice lines. We produced an F_2_ population derived from a cross between Koshihikari and ST-1 and measured each organ (panicle length, panicle rachis length, and primary branch length) in 96 of these plants. QTL analysis identified two major QTLs for panicle length (with a log_10_ odds [LOD] score >3.0) on chromosomes 5 and 6 (Fig. 2c). Interestingly, we also detected the QTL related to panicle rachis length on chromosomes 5 and the QTL related to primary branch length on chromosomes 6; these QTLs were detected in the same position. The QTL on chromosomes 5, *qPANICLE RACHIS LENGTH5* (*qPrl5*), regulates panicle rachis length. The QTL on chromosomes 6, *qPRIMARY BRANCH LENGTH6* (*qPbl6*), regulates primary branch length. These results indicate that panicle length is regulated independently by *qPrl5* and *qPbl6*.

### Isolation and characterization of Pbl6

**Fig. 3:**
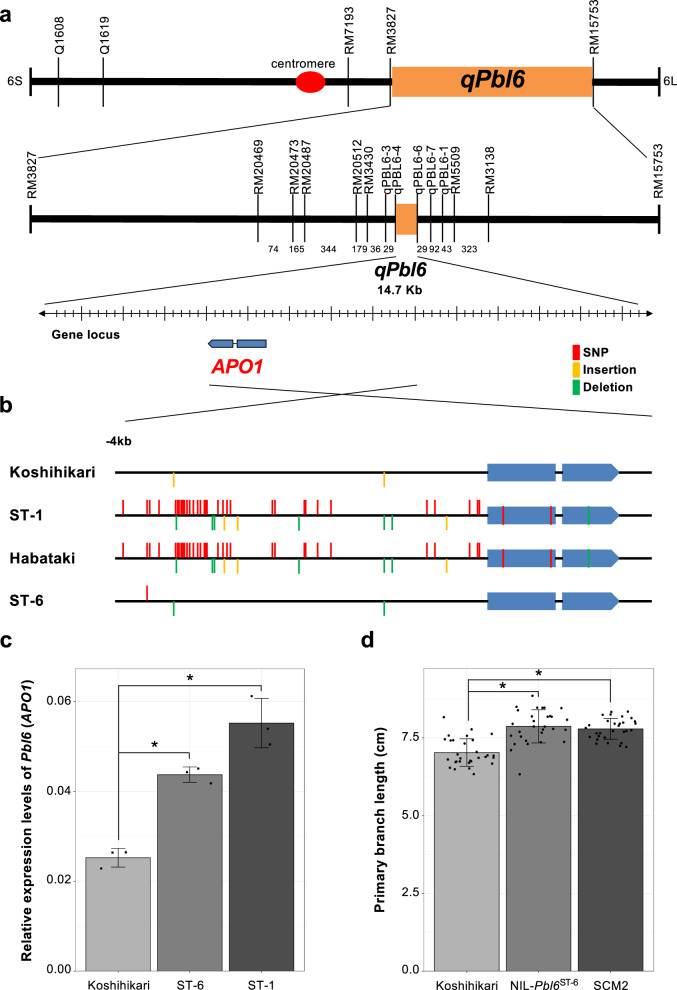
Isolation and characterization of *Pbl6*. **a** The *qPbl6* locus was detected between RM3827 and RM15753 on Chromosome 6. Numbers on the map indicate the number of recombinants. Positional cloning narrowed the *qPbl6* locus to a 14.7-kb region between qPBL6-4 and qPBL6-6 using 2475 plants. Only one gene was predicted to be located in this region by RAP-DB. **b** Sequence differences between Koshihikari, ST-1, Habataki, and ST-6 around the *APO1* region. **c** Relative expression of *Pbl6* (*APO1*) during the primary branch initiation stage by quantitative RT-PCR. Relative expression levels were calibrated based on *Ubiquitin* expression. **d** Comparison of primary branch length between Koshihikari, NIL-*Pbl6*^ST-6^ and SCM2. *n* = 3 in **c**. *n* = 30 plants in **d**. Error bars represent means ± SD. *Significant at the 5% level (Tukey’s significant difference test).

To identify the causal gene for *qPbl6*, we narrowed down the candidate region to a 14.7 kb between markers qPBL6_4 and qPBL6_6 using an F_3_ population derived from the cross between Koshihikari and ST-1. We identified only one gene, *Os06g0665400*, which was annotated as *APO1* encoding an F-box-containing protein (Fig. 3a). The *APO1* allele of indica rice variety, Habataki, increases the diameter of internodes and spikelet number^4^. The sequence of *APO1* in ST-1 completely corresponds with that of the dominant Habataki allele (Fig. 3b). We compared the expression levels of *APO1* in inflorescence meristems. *APO1* was expressed at higher levels in ST-1 compared to Koshihikari during the primary branch initiation stage (Fig. 3c). In addition, we examined primary branch length using *Strong Culm2* (*SCM2*), a near-isogenic line described in Ookawa et al.^4^, by introgressing the Habataki allele into Koshihikari. SCM2 had longer primary branches than Koshihikari (Fig. 3d). These results indicate that *Pbl6* encodes APO1 and that this gene regulates primary branch elongation.

### Isolation and characterization of Prl5

**Fig. 4:**
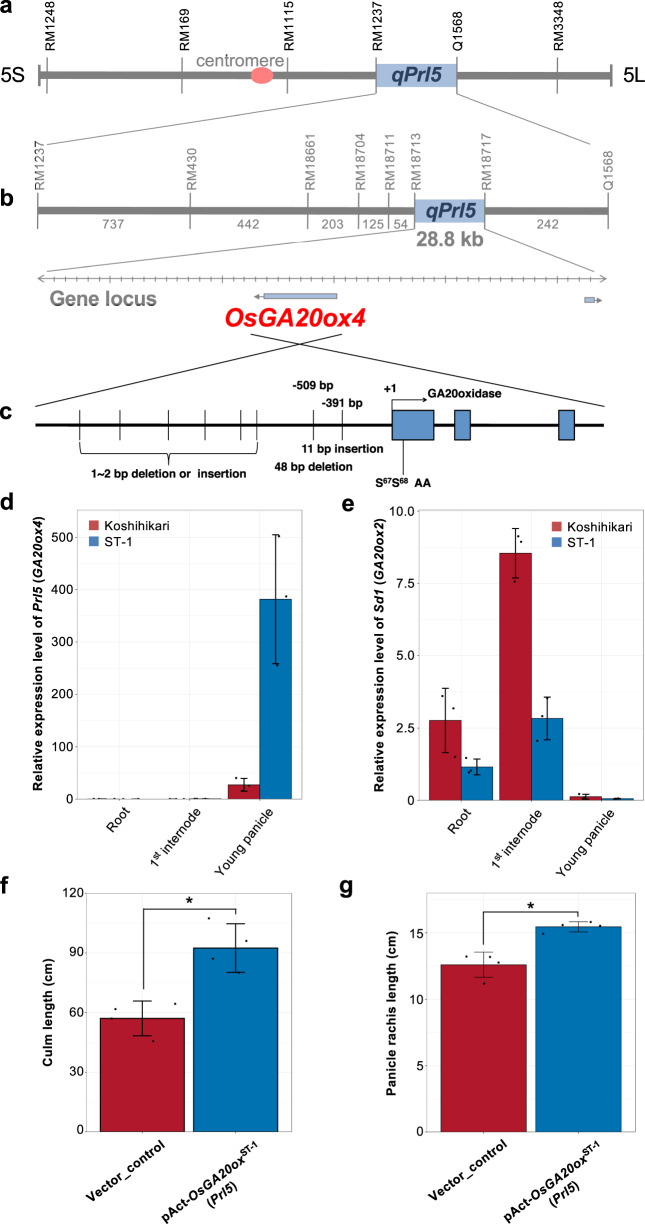
Isolation and characterization of *Prl5*. **a** The *qPrl5* locus was detected between RM1237 and Q1568 on chromosome 5. **b** Numbers on the map indicate the number of recombinants. Positional cloning narrowed the *qPrl5* locus to a 28.8-kb region between RM18713 and RM18717 using 1900 plants. Two genes were predicted to be located in this region by RAP-DB. **c** Sequence differences between Koshihikari and ST-1 around the *OsGA20ox4* region. **d**, **e** Expression analysis of *Prl5* (*GA20ox4*) (**d**) and *Sd1* (*GA20ox2*) (**e**) in roots, first internodes, and young panicles. Relative expression levels were calibrated based on *Ubiquitin* expression. Error bars represent means ± SD (*n* = 3). **f**, **g** Comparison of culm length (**f**) and panicle rachis length (**g**) between plants transformed with an empty vector (vector control) and expression vector for *OsGA20ox4*^*ST-1*^ under the control of the *Actin* promoter. Error bars represent means ± SD (*n* = 4 plants). *Significant at the 5% level (Student’s *t*-test).

We then attempted to isolate the causal gene for *qPrl5* by positional cloning. Analysis using F_3_ plants narrowed the candidate region to a 28.8 kb area between RM18713 and RM18717 (Fig. 4a, b). The Rice Annotation Project Database (RAP-DB) contains two predicted genes, *Os05g0421900* and *Os05g0422200*, in this region. *Os05g0422200* encodes a conserved hypothetical protein, and *Os05g0421900* encodes *Oryza sativa* gibberellin 20 oxidase-4 (*OsGA20ox4*). This gene is homologous to *OsGA20ox2*, also known as *SEMIDWARF1* (*Sd1*); its null allele produces a semi-dwarf phenotype and was selected during the Green Revolution^12,13^. Despite being a homologous gene of a famous green revolution gene, the function of *OsGA20ox4* has remained unclear. Because the effect of gibberellins (GAs) on cell proliferation is closely involved in the elongation of various plant organs, we focused on *OsGA20ox4*.

First, we compared the sequence of the 12 kb candidate region between the two varieties and identified several deletions and insertions in both the promoter and coding regions (Fig. 4c). GA20ox enzymes catalyze several reactions in the GA biosynthetic pathway to produce precursors that are further converted into bioactive GAs by subsequent enzymes in the pathway^14^. To investigate whether the single nucleotide variants in the coding region of this gene affect enzymatic activity, we compared the enzymatic activities of recombinant GA20OX4^Koshihikari^ and GA20OX4^ST-1^ proteins in the GA biosynthesis pathways. Both proteins showed similar levels of enzymatic activity in the GA biosynthesis pathways (Supplementary Fig. 1). This result indicates that the single nucleotide variants in the *GA20ox4*^*ST-1*^ coding region are not responsible for the high GA biosynthetic activity in ST-1. We further investigated the expression patterns of *OsGA20ox4* to confirm the notion that the base substitution in the *GA20ox4*^*ST-1*^ promoter region is responsible for the longer panicle rachis phenotype of ST-1. Quantitative RT-PCR analysis of *OsGA20ox4* in young panicles revealed that this gene was expressed at higher levels in ST-1 than in Koshihikari (Fig. 4d). *OsGA20ox4* was expressed at low levels in roots and internodes and at high levels in young panicles. These results suggest that the increased expression of *OsGA20ox4* in young panicles promotes panicle rachis elongation in ST-1. To confirm that *OsGA20ox4* is the gene underlying the *Prl5* QTL, we cloned a 1.3 kb cDNA fragment of *OsGA20ox4*, including the coding region of *OsGA20ox4*^ST-1^ (pAct::*OsGA20ox4*^ST-1^), and transformed this genomic fragment into Nipponbare. *OsGA20ox4* was expressed at high levels in the transgenic lines (Supplementary Fig. 2). The transgenic lines had a significantly higher culm length than the vector control (Fig. 4f). This result indicates that *GA20ox4*^*ST-1*^ also has enzymatic activity in vivo. The transgenic lines also had longer panicle rachises than the vector control (Fig. 4g), suggesting that *Prl5* encodes OsGA20ox4, which regulates panicle rachis elongation. The increased expression of *Prl5* presumably leads to higher GA accumulation in inflorescence meristems, resulting in panicle rachis elongation.

### Expression analysis of Prl5 and Pbl6

To clarify the different functions of the homologous genes, *Sd1* and *Prl5*, we compared their expression patterns. *Sd1* was strongly expressed in internodes, with lower expression levels in ST-1 than in Koshihikari (Fig. 4e). *Prl5* was highly expressed in young panicles, with higher expression levels in ST-1 (Fig. 4d). Although *Sd1* and *Prl5* are homologous genes, these organ-specific expression patterns contribute to their different functions. Furthermore, in ST-1, the expression level of *Sd1* was lower but expression level of *Prl5* was higher compared to Koshihikari. These results correspond with the phenotypes of ST-1, with a shorter culm length but longer panicles compared to Koshihikari (Fig. 2a, b).

**Fig. 5:**
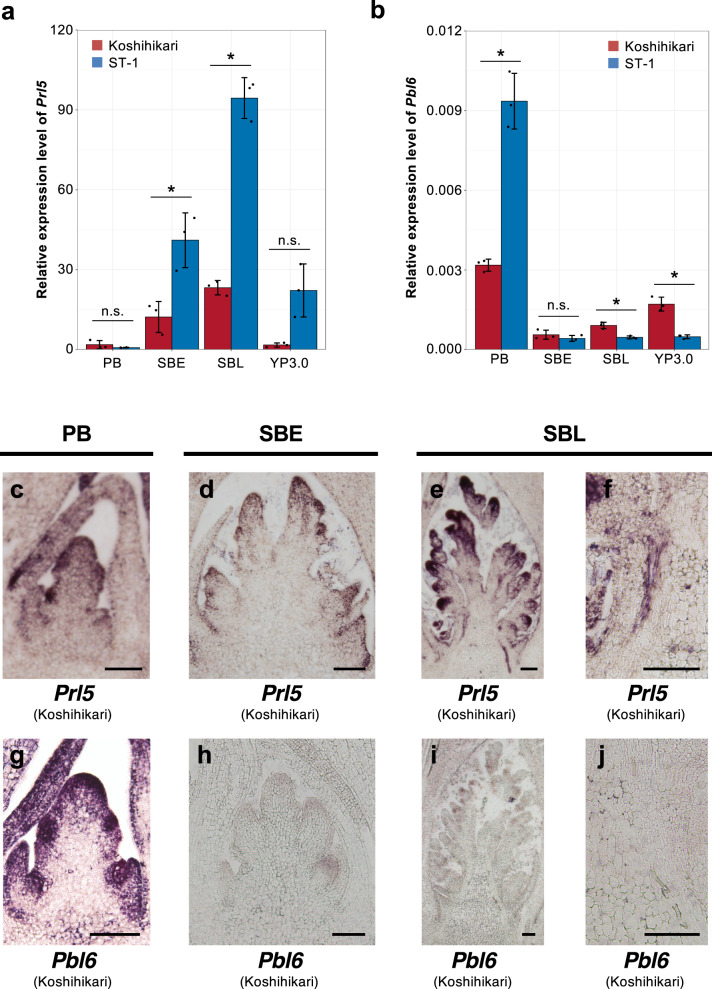
Expression analysis of *Prl5* and *Pbl6*. **a**, **b** Expression analysis of *Prl5* (**a**) and *Pbl6* (**b**) in inflorescence tissue at various developmental stages. PB primary branch initiation stage, SBE secondary branch initiation earlier stage, SBL secondary branch initiation later stage, YP3.0: 3 mm stage of young panicles. Relative expression levels were calibrated based on *Ubiquitin* expression (**a**, **b**). Error bars represent means ± SD (*n* = 3). *Significant at the 5% level (Student’s *t*-test). **c**–**j** In situ hybridization of *Prl5* (**c**–**f**) and *Pbl6* (**g**–**j**) during panicle development in Koshihikari. Panels **c** and **g** are at the stage of primary branch differentiation. Panels **d** and **h** are at the earlier stage of secondary branch differentiation. Panels **e** and **i** are at the later stage of secondary branch differentiation. Panels **f** and **j** are close-up views of vascular bundles at the later stage of secondary branch differentiation. Scale bars: 100 μm.

Because differences in panicle length may appear during the early stages of panicle development, we investigated *Prl5 and Pbl6* expression levels during several stages of panicle development. The expression of *Prl5* was higher in ST-1 vs. Koshihikari during the secondary branch initiation stage and the 3 mm stage (Fig. 5a). The expression level of *Prl5* was highest during the later stage of secondary branch initiation; this trend was observed in both varieties. By contrast, the expression level of *Pbl6* was higher in ST-1 than in Koshihikari during the primary branch initiation stage (Fig. 5b). The expression level of *Pbl6* was higher during the primary branch initiation stage compared to the other stages; this trend was observed in both varieties. *Pbl6* expression was activated first, followed by *Prl5* expression. There were major differences in the timing of expression between *Prl5* and *Pbl6*. *Pbl6* was expressed at the earlier stages of panicle development. A high expression level of *Pbl6* increases the size of the inflorescence meristem^5^, resulting an elongated primary branch length. By contrast, *Prl5* were expressed at later stages.

We also examined the temporal and spatial expression patterns of these genes by in situ hybridization analyses of inflorescences at various developmental stages (Fig. 5c–j). *Pbl6* expression was detected near the primary branch meristems in Koshihikari (Fig. 5g–j). *Prl5* expression was detected near the primary and secondary branch meristems (Fig. 5c–e) and vascular bundles (Fig. 5e, f) in Koshihikari. *SoGA20ox1*, a homolog of *Prl5*, is known to be associated with stem elongation in spinach under long-day (LD) conditions^15,16^. *SoGA20ox1* transcripts were also detected in vascular tissues by in situ hybridization^17^, which is similar to the current results. This suggests that the expression in the vascular bundle is important for regulating panicle rachis elongation.

### Effect of Prl5 and Pbl6 on panicle architecture

**Fig. 6:**
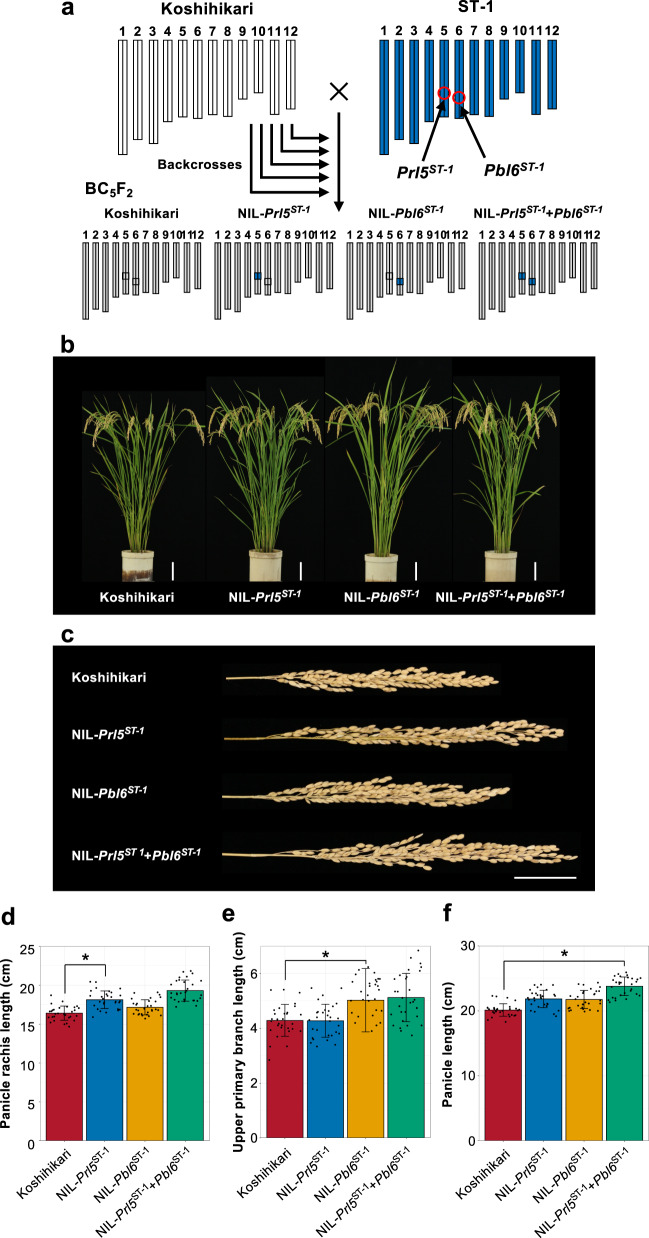
Effects of the two genes on panicle architecture under field conditions. **a** Graphical genotypes of BC_5_F_2_ plants derived from a cross between Koshihikari and ST-1. White and blue bars indicate Koshihikari and ST-1 chromosomes, respectively. Red circles on ST-1 indicate the positions of *Prl5*^ST-1^ and *Pbl6*^ST-1^. **b** Gross morphologies of the NILs. Scale bar: 10 cm. **c** Panicle morphologies of the NILs. Scale bar: 5 cm. **d**–**f** Comparison of panicle traits. **d** Panicle rachis length. **e** Lengths of the three upper primary branches. **f** Panicle length. Error bars represent means ± SD (*n* = 30 plants). *Significant at the 5% level (Tukey’s significant difference test).

As *Prl5* and *Pbl6* leads to increased panicle length, which could greatly alter panicle architecture, we evaluated the effects of the *Prl5* and *Pbl6* loci from ST-1 on panicle architecture. Based on the mapping results, we developed NILs from BC_5_F_2_ populations that contained the *Prl5* and *Pbl6* region from ST-1 in the Koshihikari background (Fig. 6a). The NILs did not differ from one another with respect to plant morphology (Fig. 6b), but their panicle morphology clearly differed (Fig. 6c). We conducted detailed analysis of the panicles of these NILs. The panicle rachises of NIL-*Prl5*^ST-1^ were longer than those of Koshihikari (Fig. 6d). The primary branches of NIL-*Pbl6*^ST-1^ were longer than those of Koshihikari (Fig. 6e). Due to the effects of *Prl5* and *Pbl6*, the panicles of NIL-*Prl5*^ST-1^ + *Pbl6*^ST-1^ were 3.7 cm longer than those of Koshihikari (Fig. 6f).

**Fig. 7:**
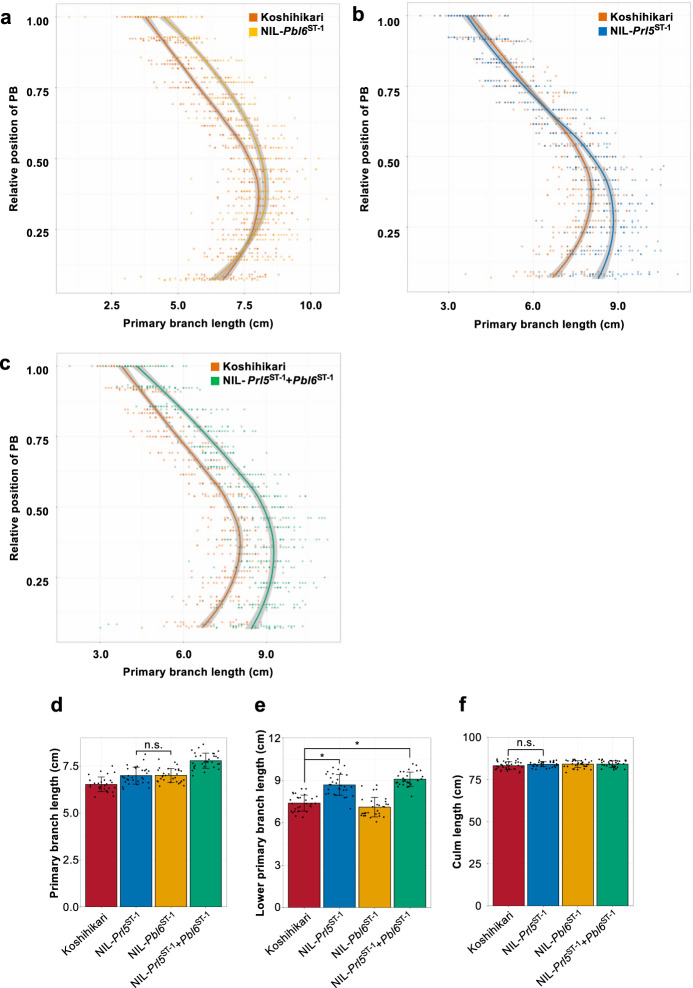
Effects of the two genes on primary branch elongation. **a**–**c** Comparison of every primary branch length. Solid lines show regression curves. Orange line and dots indicate Koshihikari. Yellow line and dots indicate NIL-*Pbl6*^ST-1^ (**a**). Blue line and dots indicate NIL-*Prl5*^ST-1^ (**b**). Green line and dots indicate NIL-*Prl5*^ST-1^ + *Pbl6*^ST-1^ (**c**). *n* = 42 plants. **d**–**f** Comparison of panicle traits and culm length. **d** every primary branch length. **e** Lower three primary branch length. **f** Culm length. Error bars represent means ± SD (*n* = 30 plants in **d** and **e**, *n* = 35 plants in **f**). *Significant at the 5% level (Tukey’s significant difference test).

Panicle length includes panicle rachis length and the length of the top primary branch. Thus, in the current study, we focused only on upper primary branch length. However, rice plants have many primary branches from the lower to upper axis. Therefore, we investigated whether the effect of *Pbl6* is limited to upper primary branch elongation or if it affects the elongation of every primary branch. We observed every primary branch length from lower to upper axis. The Local Regression (LOESS) curves, which represent the length of every primary branch in all four lines, indicate that *Pbl6* has an effect on upper primary branch elongation (Fig. 7a). Interestingly, this result also suggests that *Prl5* has an effect on lower primary branch elongation (Fig. 7b). In fact, there was no difference in the average length of any primary branch length between NIL-*Prl5*^ST-1^ and NIL-*Pbl6*^ST-1^ (Fig. 7d). The lower primary branches of NIL-*Prl5*^ST-1^ and NIL-*Prl5*^ST-1^ + *Pbl6*^ST-1^ were longer than those of Koshihikari and NIL-*Pbl6*^ST-1^ (Fig. 7e). Due to the combination of these two genes, every primary branch from the lower to upper axis was longer in these lines than in Koshihikari (Fig. 7c).

Detailed observation of panicle architecture revealed that *Prl5* affects panicle rachis and lower primary branch elongation, whereas *Pbl6* affects upper primary branch elongation. *Prl5* was expressed during the secondary branch initiation stage. Higher GA levels in vascular bundles led to panicle rachis elongation (Fig. 5a, e, f). *Prl5* was expressed in the vascular bundles of young panicles, which are located near the lower primary branch meristems, indicating that bioactive GAs promote not only the elongation of the panicle rachis but also the lower primary branches. By combining *Prl5* and *Pbl6* in various ways, we could design plants with panicle architecture like that shown in Fig. 1c–f. Because the morphogenesis of multiple organs occurs simultaneously in parallel during panicle development, *Prl5* and *Pbl6* affect multiple panicle organs. However, when considering only panicle length, we confirmed that *Prl5* and *Pbl6* regulate panicle length independently.

We then evaluated *Prl5* expression in the NIL. The expression level of *Prl5* was higher in the young panicles of NIL-*Prl5*^ST-1^ vs. Koshihikari (Supplementary Fig. 3a). We also examined *Prl5* expression during the later stage of secondary branch initiation by in situ hybridization (Supplementary Fig. 4). We confirmed the expression patterns of *Prl5* in the primary and secondary branch meristems and vascular bundles of all four lines. The expression patterns of NILs were well corresponded with that of Koshihikari. As expected, the expression level of *Prl5* in vascular bundles was stronger in NIL-*Prl5*^ST-1^ and NIL-*Prl5*^ST-1^ + *Pbl6*^ST-1^ than in Koshihikari and NIL-*Pbl6*^ST-1^. To confirm the organ-specific expression of *Prl5*, we compared the expression patterns of *Prl5* and *Sd1* in NIL-*Prl5*^ST-1^ (Supplementary Fig. 3)*. Prl5* was expressed at higher levels in young panicles than in internodes. By contrast, *Sd1* was expressed at higher levels in the internodes of both lines. There was no difference in the expression level of *Sd1* between NIL-*Prl5*^ST-1^ and Koshihikari. In fact, there was no difference in culm length (Fig. 7f). These results suggest that *Prl5* does not affect plant architecture, but it affects only panicle architecture.

Finally, to confirm that the increased expression of *Prl5* in young panicles leads to the increased accumulation of bioactive GA, we quantified GA levels in Koshihikari and NIL-*Prl5*^ST-1^ (Supplementary Fig. 5). The levels of both GA_44_ and GA_20_ were reduced in NIL-*Prl5*^ST-1^. In addition, GA_53_ and GA_24_ levels appeared to be reduced in NIL-*Prl5*^ST-1^, but these differences were not significant. These results suggest that *Prl5* contributes to the increased accumulation of bioactive GA species GA_1_ and GA_4_ during panicle development (see Supplementary Note for detailed discussion).

### Gene expression analysis of the NILs

**Fig. 8:**
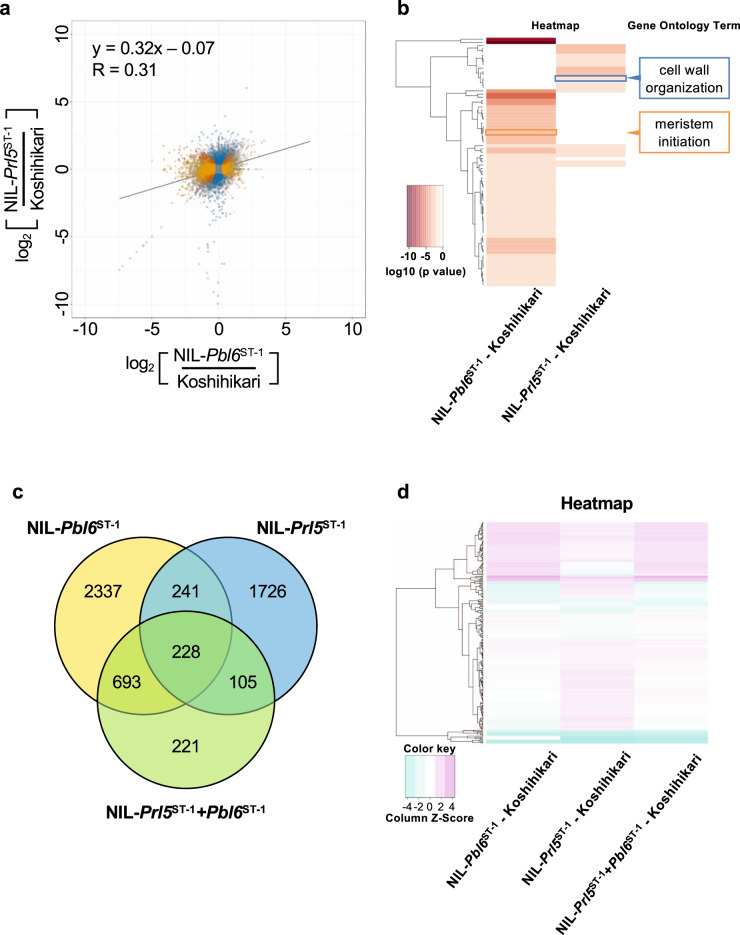
Comparison of the gene expression patterns of the NILs. **a** RNA was extracted from 2-mm stage young panicles of Koshihikari, NIL-*Prl5*^ST-1^, NIL-*Pbl6*^ST-1^, and NIL-*Prl5*^ST-1^ + *Pbl6*^ST-1^ and used for RNA sequencing. The *y* axis shows the log2 ratios of the changes in gene expression between Koshihikari and NIL-*Prl5*^ST-1^, whereas the *x* axis shows the log2 ratios of the changes in gene expression between Koshihikari and NIL-*Pbl6*^ST-1^. The solid line represents the regression line. *y* = 0.32 X −0.07, *R* = 0.31. Yellow dots indicate genes whose expression levels changed in NIL-*Pbl6*^ST-1^ compared with Koshihikari. Blue dots represent genes whose expression levels changed in NIL-*Prl5*^ST-1^ compared with Koshihikari. Orange dots represent common genes whose expression levels changed in both NIL-*Prl5*^ST-1^ and NIL-*Pbl6*^ST-1^. Gray dots represent other genes. **b** Heatmap show the *P*-value (*P* < 0.05 cutoff) significance of GO terms for *Prl5* and *Pbl6* target genes. The GO terms listed are the characteristic enriched biological process GO terms for each *Prl5* (blue) and *Pbl6* (orange). The complete GO term lists and their significance levels are given in Supplementary Data 3. **c** Venn diagram of genes upregulated or downregulated in NIL-*Prl5*^ST-1^ (shown in the blue circle), NIL-*Pbl6*^ST-1^ (shown in the yellow circle), and NIL-*Prl5*^ST-1^ + *Pbl6*^ST-1^ (shown in the green circle). **d** Heatmap of the expression patterns of the 228 common genes. The expression levels of these 228 genes changed in all three NILs compared with Koshihikari. The expression level of each gene compared with expression in Koshihikari is shown on the map. Red and blue indicate higher and lower expression, respectively. A color scale is shown at the bottom.

To confirm the effects of *Prl5* and *Pbl6* on panicle length at the gene expression level, we performed RNA-seq analysis to compare the expression patterns of these genes in young panicles of the NILs. The results are shown in Fig. 8a, where the *y* axis represents the changes in gene expression between Koshihikari and NIL-*Prl5*^ST-1^ and the *x* axis represents the changes in gene expression between Koshihikari and NIL-*Pbl6*^ST-1^ in terms of log2 values. The correlation coefficient was 0.31, suggesting that *Prl5* and *Pbl6* have different biological effects on gene expression. Therefore, *Prl5* and *Pbl6* also regulate panicle length independently at the gene expression level. Gene Ontology (GO) representation analysis indicated that the biological functions of the two genes are quite different (Fig. 8b, Supplementary Data 3). GO terms involved in cell wall organization (GO:0071555) was enriched, implying that *Prl5* closely related to cell elongation of vascular bundle in young panicle. Consistent with *Pbl6*’s role, GO terms associated with meristem initiation (GO:0010014) was enriched. These results also strongly suggest that *Prl5* and *Pbl6* regulate panicle length independently.

RNA-seq analysis revealed 5551 differentially expressed genes in NIL-*Prl5*^ST-1^, NIL-*Pbl6*^ST-1^, and NIL-*Prl5*^ST-1^ + *Pbl6*^ST-1^ compared to Koshihikari (Fig. 8c, Supplementary Data 1 and 2). *Prl5* regulates 1726 genes and *Pbl6* regulates 2337 genes independently. It is highly likely that the 228 common genes with differential expression patterns between Koshihikari and other NILs are closely associated with panicle rachis and primary branch elongation. To compare the expression patterns of the 228 common differentially expressed genes, we constructed a heatmap (Fig. 8d). Several genes had the same expression patterns in all three NILs, including *BETA-EXPANSIN7* and *CELLULOSE SYNTHASE LIKE F2*, which have important roles in cell elongation. Furthermore, the expression patterns of these genes in NIL-*Prl5*^ST-1^ and NIL-*Pbl6*^ST-1^ were quite different, whereas those in NIL-*Pbl6*^ST-1^ and NIL-*Prl5*^ST-1^ + *Pbl6*^ST-1^ were quite similar. Unexpectedly, among the 228 common genes, *Pbl6* appeared to function upstream of *Prl5* at the gene expression level. Expression analysis of Koshihikari and ST-1 revealed that *Pbl6* is expressed first, followed by *Prl5* (Fig. 5a, b). *Pbl6* has stronger effects on panicle development in NIL-*Prl5*^ST-1^ + *Pbl6*^ST-1^, as it was expressed at an earlier stage of panicle development. The differences in the timing of the highest expression levels of *Prl5* and *Pbl6* result in the similar expression patterns of the 228 common genes in NIL-*Pbl6*^ST-1^ and NIL-*Prl5*^ST-1^ + *Pbl6*^ST-1^.

### Effects of Prl5 and Pbl6 on yield performance

**Fig. 9:**
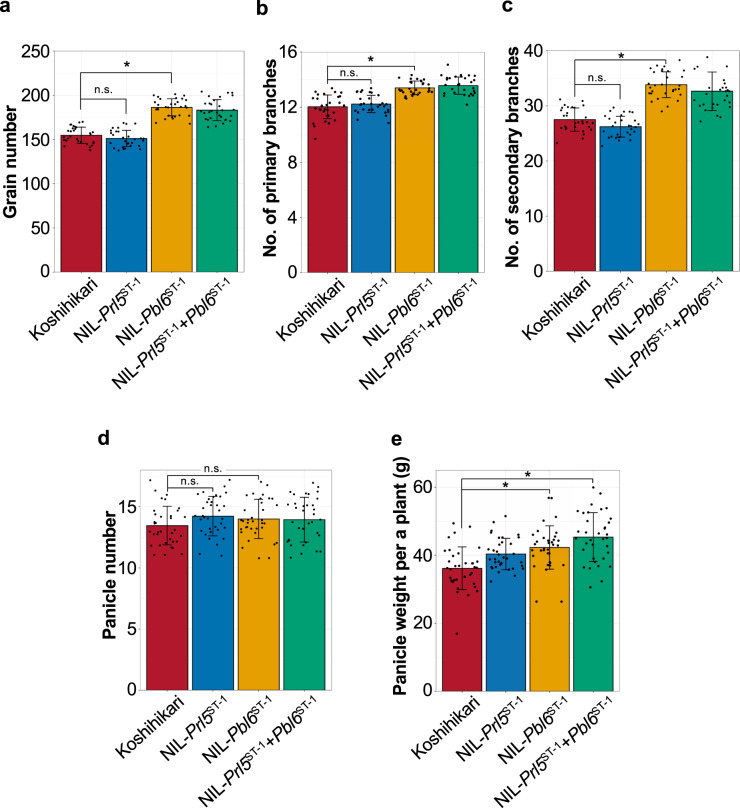
Effects of two genes on yield performance under field conditions. **a**–**e** Comparison of yield traits. **a** Grain number. **b** Number of primary branches. **c** Number of secondary branches. **d** Panicle number. **e** Panicle weight per plant. *n* = 30 plants in **a**–**c**; *n* = 35 plants in **d**, **e**. Error bars represent means ± SD. *Significant at the 5% level (Tukey’s significant difference test).

We further evaluated the effects of *Prl5* and *Pbl6* on yield performance under filed condition. NIL-*Prl5*^ST-1^ showed no obvious effect on panicle branching (Fig. 9a–c). As *Prl5* does not have pleiotropic effects, we could alter only panicle length by introducing *Prl5* while maintaining variation-specific branching numbers. NIL-*Pbl6*^ST-1^ produced more spikelets per panicle than Koshihikari due to an increase in branch number (Fig. 9a–c). *Pbl6* had pleiotropic effects not only primary branch length but also on panicle branching. Although both *Prl5* and *Pbl6* led to increased panicle length, there were distinct differences between the functions of these two genes. The introgression of *Prl5* and *Pbl6* had no effects on panicle number (Fig. 9d). NIL-*Pbl6*^ST-1^ showed higher yields than Koshihikari due to increased grain number per panicle. NIL-*Prl5*^ST-1^ + *Pbl6*^ST-1^ had the highest yield among lines (Fig. 9e). These results suggest that combining *Prl5* with a gene related to panicle branching and total yields, such as *Pbl6*, represents an efficient method for breeding higher yielding rice varieties.

*GNP1* (*grain number per panicle1*) encodes *OsGA20ox1*^18^. Wu et al. proposed that the upregulation of *GNP1/GA20ox1* in the inflorescence meristems increases cytokinin activity via KNOX-mediated feedback regulation and increases GA catabolic activity. As a result, this process increases cytokinin activity, thereby rebalancing cytokinin and GA activity and increasing grain number and grain yield. However, in the current study, the increased expression of *Prl5* did not affect grain number (Fig. 9a), perhaps due to the expression levels and temporal expression patterns of other genes. The expression level of *GNP1/GA20ox1* was much higher than that of *Sd1/GA20ox2* and *Prl5/GA20ox4* in both Koshihikari and NIL-*Prl5*^*ST-1*^ (Supplementary Fig. 6a). In addition, the expression patterns of four *KNOX* genes in panicle meristems, including *OSH1*, *OSH6*, *OSH15*, and *OSH71*, did not differ between Koshihikari and NIL-*Prl5*^*ST-1*^ (Supplementary Fig. 6b). Consequently, the levels of several endogenous cytokinins and cytokinin biosynthesis intermediates did not differ between Koshihikari and NIL-*Prl5*^*ST-1*^ (Supplementary Fig. 7). These results suggest that moderately strong expression of *Pr15/GA20ox4* with no induction of *KNOX* genes or active cytokinin production affects panicle rachis length but not grain number. An effect on length but not branching is a key feature of *Prl5/GA20ox4*.

### Isolation and characterization of novel Pbl6 allele

Our analysis showed the possibility that panicle architecture greatly alters depending on the expression levels of *Prl5* and *Pbl6*. To confirm genetically whether these two genes can generate tremendous morphological diversity in rice panicle, we explored novel alleles of two genes and investigated the relationship between expression levels and panicle phenotypes. Koshihikari and ST-6, which have longer panicle length next to ST-1, were chosen for parental varieties (Fig. 1i). We produced F_2_ population derived a cross between Koshihikari and ST-6 and measured panicle phenotypes in these plants. QTL analysis identified one QTL for primary branch length on chromosomes 6. Positional cloning narrowed the candidate region and identified only one gene, *Pbl6*. The sequence of *Pbl6* in ST-6 is different from the Habataki and ST-1 allele (Fig. 3b). The expression level of *Pbl6* in ST-6 was higher than Koshihikari but lower than ST-1 (Fig. 3c). Using BC_5_F_2_ population generated from the cross between Koshihikari and ST-6, NIL carrying the ST-6 *Pbl6* allele was selected. NIL-*Pbl6*^ST-6^ had longer primary branches than koshihikari (Fig. 3d). These results indicate that *Pbl6* regulates primary branch length also in ST-6. Since there was only a difference in the promoter region between the sequence of *Pbl6* in Koshihikari and ST-6, we concluded that the effect on the primary branch length is due to the different expression level. There was no difference in the upper primary branch length between NIL-*Pbl6*^ST-6^ and Koshihikari (Supplementary Fig. 8a). However, the Local Regression (LOESS) curves indicate that, consistent with the expression levels (Fig. 3c), the effect of *Pbl6*^ST-6^ is moderate compare to that of *Pbl6*^ST-1^ (Supplementary Fig. 8b). These results suggest that *Pbl6*^ST-6^ is a different allele, which has moderate effects on primary branch elongation. We further evaluated the effect of *Pbl6*^ST-6^ on yield performance under field condition. The NIL-*Pbl6*^ST-6^ produced more spikelets per panicle than koshihikari due to an increase in branch number (Supplementary Fig. 8c–e). In addition, the effect of *Pbl6*^ST-6^ on panicle branching was moderate compare to *Pbl6*^ST-1^. These results suggest that *Pbl6*^ST-6^ is a different allele of *Pbl6* and primary branch length is certainly modulated by the expression levels of *Pbl6*.

**Fig. 10:**
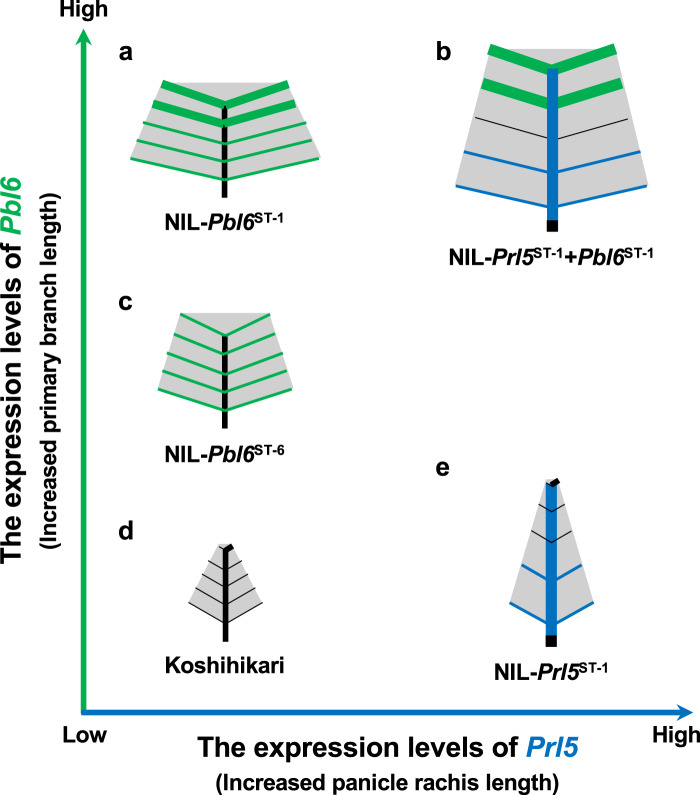
Various panicle architectures are generated by *Prl5* and *Pbl6*. Blue lines indicate the effects of *Prl5* and green lines indicate the effects of *Pbl6*.

In this study, we found two genes, *Prl5* and *Pbl6*, independently regulate panicle length. We further identified novel *Pbl6* allele, *Pbl6*^ST-6^. Combining these genes, we designed numerous panicle architecture (Fig. 10). Although further exploring novel alleles will be needed, we concluded that many of various panicle architecture in rice are generated by various combinations of *Prl5* and *Pbl6* alleles.

## Discussion

Previous studies of rice panicle architecture have primarily focused on panicle branching, which directly affects grain yield. However, panicle length also has a key role in yield improvement. Increasing only panicle branching leads to plants with high grain density that tend to suffer from panicle diseases, such as false smut^19^. Therefore, reducing grain density by regulating panicle and branch length is important for reducing yield losses, but the underlying regulatory mechanisms have been largely unknown. In the current study, we identified two QTLs that independently increase panicle length: *PANICLE RACHIS LENGTH5* (*Prl5*), encoding Gibberellin 20 oxidase; and *PRIMARY BRANCH LENGTH6* (*Pbl6*), encoding APO1. Transcriptome analysis revealed that these two genes independently regulate panicle length at the gene expression level. Using various combinations of *Prl5* and *Pbl6*, we designed numerous panicle architecture without any trade-off between panicle length and grain number. Designing ideal panicle architecture that considers the spatial arrangement of branches by regulating panicle length as well as branch number is essential for obtaining substantial yield improvements in future rice breeding programs.

GAs are crucial phytohormones for plant growth and developmental^20,21^. Several genes encoding GA metabolic enzymes and key factors in GA signal transduction pathways have been isolated from various plants^22^. Tenreira et al.^23^ revealed that *FveGA20ox4* dictates the flowering-runnering decision in diploid strawberry, suggesting that GAs have roles in altering plant architecture and plant diversity. There are four GA20ox genes in the rice genome, two of which (*OsGA20ox1* and *OsGA20ox2*) are identical to previously reported genes. In addition, we revealed a function for *OsGA20ox4*.

*Sd1* (*SEMIDWARF1*) encodes *OsGA20ox2*; its null allele exhibits a semi-dwarf phenotype but causes no obvious defects in other agronomic traits. This gene was widely used to breed lodging-resistant rice plants during the Green Revolution. Although *Sd1/GA20ox2* was strongly expressed in internodes, *Pr15/GA20ox4* was expressed at low levels in roots and internodes but highly expressed in young panicles (Fig. 4d, e). Although *Sd1/GA20ox2* and *Pr15/GA20ox4* are homologous genes, these genes were specifically expressed in internodes and young panicles, respectively. These organ-specific expression patterns contribute to the different functions of these genes. Although *Sd1/GA20ox2* was expressed at a lower level in ST-1 vs. Koshihikari, *Pr15/GA20ox4* was expressed at a higher level in ST-1. These expression patterns closely correspond to the desirable plant morphology of ST-1 for agriculture, including its shorter culms and longer panicles (Fig. 2a, b).

*GNP1* (*Grain Number Panicle 1*) encodes *OsGA20ox1*, which increase grain number. Although both *GNP1*/*GA20ox1* and *Prl5*/*GA20ox4* expressed in young panicles, our results indicate that the functions of two genes were different (Fig. 9a, Supplementary Figs. 6 and 7). This difference would result from the expression patterns. The expression level of *Prl5*/*GA20ox4* was lower than that of *GNP1*/*GA20ox1*, but the moderate high expression of *Prl5*/*GA20ox4* was enough for panicle rachis elongation. The expression pattern of *Prl5*/*GA20ox4*, in the vascular bundles at the secondary branch initiation stage, could contribute to regulate panicle rachis length.

*APO1* has pleiotropic effects on rice, as it simultaneously enhances culm strength and increases spikelet number^4,5^. Our findings reveal a novel effect of *APO1*. *Pbl6* increases grain number while elongating primary branches (Figs. 6e and 9a–c). As a result, introducing *Pbl6* did not have a negative effect on the density of branches on each primary branch (Supplementary Fig. 9). Thus, both *Prl5* and *Pbl6* help maintain the proper spatial arrangement of grains. Although both *Prl5* and *Pbl6* regulate panicle length, there are obvious differences in the functions of these two genes.

*DEP1* improves yield potential by reducing panicle internode length while increasing grain number^9^. As a result, introducing *DEP1* into rice increases panicle density. *DEP1* has already been incorporated into Chinese rice breeding. However, Japanese breeders have not used *DEP1* for rice breeding due to frequent rains, especially during the rice-cropping season. The high humidity increases the growth of fungi, which cause many diseases. Plants with erect, dense panicles are poorly ventilated, facilitating the growth of fungi. Therefore, rice plants with dense panicles are not suitable for growth in countries with humid seasons such as Japan.

Unlike *DEP1*, *Prl5* and *Pbl6* are useful for maintaining the appropriate density of panicle branches. By combining *Prl5* and *Pbl6* in various ways, we designed five patterns of panicle architecture (Fig. 10). The introduction of *Prl5*^*ST-1*^ helped maintain low primary branch density in NIL-*Prl5*^*ST-1*^, as shown in Fig. 10e. The introduction of *Pbl6*^*ST-1*^ helped maintain a low density of secondary rachilla in NIL-*Pbl6*^*ST-1*^, as shown Fig. 10a. The introduction of *Pbl6*^*ST-6*^ moderately helped maintain a low density of secondary rachilla in NIL-*Pbl6*^*ST-6*^, as shown Fig. 10c. Both *Prl5*^*ST-1*^ and *Pbl6*^*ST-1*^ helped maintain an appropriate spatial arrangement of grains in *Prl5*^*ST-1*^ + *Pbl6*^*ST-1*^ by elongating panicle rachis length and primary branch length, as shown in Fig. 10b. The panicle architecture of NIL-*Prl5*^*ST-1*^ + *Pbl6*^*ST-1*^ is suitable for high-humidity cultivation and has high yields due to the additive effects of *Pbl6*^*ST-1*^.

We further identified different *Pbl6* allele, *Pbl6*^ST-6^. This result suggests that there are various alleles of *Pbl6* in natural rice varieties. The differences in *Pbl6* expression levels determine primary branch length of each varieties and generate its diversity. It is assumed that diverse panicle rachis length result from various *Prl5* alleles. Our findings showed the first example that combinations of two genes generate diverse panicle architecture.

Novel alleles and additional genes that regulate panicle phenotypes must be identified in order to design ideal panicle architecture for each cultivation area. In addition, our findings indicate that combining genes related to panicle branching and panicle length is a useful approach for improving crop productivity. Several genes have already been shown to increase branch number, such as *GN1a*, *WFP*, and *FZP*^2,3,6^. We believe that combining *Prl5* and *Pbl6* with these genes represents an excellent strategy for developing higher yielding rice varieties.

In this study, we produced NILs with various panicle architectures. These NILs are useful materials for rice breeding and should facilitate further research for improving yield potential and grain quality. Grain number has been a key breeding target for improving yield potential. However, simultaneously improving grain number and grain quality is a major challenge due to the negative correlation between grain number and quality. Increasing seed productions often leads to smaller, low-quality grains. Indeed, natural variants of the rice G-protein γ subunit genes *DEP1* and *GS3* have increased grain yields but typically show only mediocre grain quality^24^. Filling rate directly affects grain quality and is one of the main factors regulating yield potential. Breeders have recently begun to focus on filling rate as well as grain number. Therefore, in order to further improve yield potential, overcoming the problem of poor grain filling is an important issue that should be addressed in the future^25^. The grain-filling rate varies greatly depending on panicle position, but how the position of each grain and panicle length affect filling rate has not yet been clarified due to the lack of suitable experimental materials. By using NILs produced in the current and future studies combined with known genes such as *GN1a* and *FZP*, it should possible to investigate whether panicle length and the location of each grain affects filling rate. Such information would be useful for identifying the ideal panicle architecture for high-quality grain production to steadily improve rice productivity, as well as designing panicle architecture suitable for different environments and cultivation areas. Identifying genes related to the diversity of rice panicle architecture could provide a deeper understanding of panicle morphogenesis and help breeders create new varieties.

## Methods

### Plant materials and growth conditions

ST-1, ST-5, and ST-6 was selected from the rice collections of the Field Science Center at Nagoya University. The ST-1 × Koshihikari F_1_ plant was backcrossed five times with Koshihikari to generate near-isogenic lines. NIL-*Prl5*^ST-1^, NIL-*Pbl6*^ST-1^, and NIL-*Prl5*^ST-1^ + *Pbl6*^ST-1^ were selected from the BC_5_F_2_ population using single-sequence repeat (SSR) markers. The ST-6 × Koshihikari F_1_ plant was backcrossed five times with Koshihikari to generate near-isogenic lines. NIL-*Pbl6*^ST-6^ was selected from the BC_5_F_2_ population using SSR markers. All nontransgenic materials were grown under natural conditions in a paddy field at the Field Science Center at Nagoya University, Togo, Aichi, Japan. Seeds were germinated in a seedbed in early May and transplanted to the field in late June. Plants were grown in the paddy field by single transplanting under conventional cultivation conditions. Transgenic plants were grown in isolated greenhouses at 24 °C under a natural light environment.

### Observation of panicle architecture

A main panicle was sampled from each plant for observation. For QTL analysis, panicle length, panicle rachis length, and primary branch length were measured. To evaluate panicle architecture in the NILs, panicle length, panicle rachis length, primary branch length, number of primary branches, number of secondary branches, and total grain number were measured.

### QTL analysis and fine mapping

QTL analysis was performed with 96 F_2_ plants obtained from a cross between Koshihikari and ST-1. F_2_ recombinant plants were genotyped to identify homozygous recombinant plants for *qPrl5* and *qPbl6*, respectively. Selected F_2_ plants were selfed, and fine mapping was performed with the F_3_ populations. Also, QTL analysis was performed with 96 F_2_ plants obtained from a cross between Koshihikari and ST-6. Fine mapping was performed with the BC_6_F_2_ populations. Genomic DNA was extracted from leaves using the cetyltrimethylammonium bromide (CTAB) method, and pure DNA samples were used for genotyping using the SSR markers listed in Supplementary Table 1. PCR for genotyping was performed with Taq polymerase. Data analyses were performed using the software package R/QTL (R version 3.3.3; R/qtl package 1.41–6). QTLs were identified using Haley–Knott regression, and the significance threshold was set using 1000 permutations.

### Sequence analysis

Sequence analysis of *Prl5* was carried out by comparing the sequences of this gene in ST-1 and Koshihikari. Sequence analysis of *Pbl6* was carried out by comparing the sequences of this gene in ST-1, Habataki, ST-6, and Koshihikari. The promoter and coding regions of *Prl5* and *Pbl6* were amplified by PCR with KOD FX Neo (Toyobo) and the primers listed in Supplementary Table 2. PCR products were purified with Wizard® SV Gel and PCR Clean-Up System (Promega) and sequenced with the primers listed in Supplementary Table 2.

### RNA extraction and quantitative RT-PCR analysis

Total RNA was extracted from roots, first internodes, and inflorescence meristems as described by Sambrook et al.^26^ and treated with DNase I. First-strand cDNA was synthesized from 1 μg of total RNA using a QuantiTect Reverse Transcription Kit (Qiagen). Quantitative RT-PCR was performed using a QuantiTect SYBR Green PCR Kit (Qiagen) and a LightCycler System (LightCycler 1.5; Roche Applied Science). The relative standard curve method was used to calculate the relative transcript levels of the target genes. The results were confirmed using three independent biological replicates. For all experiments, the transcript level of each gene was normalized with that of endogenous *OsUBC32*. The primer sets used for PCR are listed in Supplementary Table 3.

### In situ hybridization

Inflorescence meristems in fixed with 4% (w/v) paraformaldehyde and 0.25% glutaraldehyde in 0.1 M sodium phosphate buffer (pH 7.2) overnight at 4 °C and embedded in SCEM compound (SECTION-LAB, Japan). The cut surface was covered with an adhesive film (Cryofilm type IIC9, SECTION-LAB, Japan), and frozen sections (8–14 μm) were prepared with a cryostat (CM 1850 Leica Microsystems, Germany) as previously described^27^. Digoxigenin-labeled anti-sense RNA probes for *Prl5* and *Pbl6* transcripts were prepared as described by Komatsu et al.^8^. Hybridization and immunological detection of the digoxigenin-labeled probes were performed as previously described with some modifications^28^. The primers used to amplify the cDNAs are listed in Supplementary Table 4.

### Generation of transgene constructs and plant transformation

To produce transgenic lines harboring *Prl5*, the coding sequence of *Prl5* was amplified from ST-1 cDNA using a forward primer (5′-GCAAGCTTATGCATGCATCTCCTCACCCATTAC-3′) containing a HindIII site and a reverse primer (5′-ATACTAGTCTAGACGGTGGCCGCCTGGGTCG-3′) containing a *Spe*I site. The fragment was cloned into the *Hin*dIII–*Spe*I sites of pAct::nos-pCAMBIA1380 and introduced into, japonica rice variety Nipponbare by *Agrobacterium tumefaciens* (EHA105)-mediated transformation as described by Ozawa et al.^29^. Transformed cells and plants were selected based on hygromycin resistance, and regenerated seedlings were grown to maturity in pots in a greenhouse. Four T_0_ plants were analyzed. Control plants were generated via transformation with empty vector.

### Measuring endogenous cytokinin and GA levels

Two-mm stage young panicles of Koshihikari and NIL-*Prl5*^ST-1^ were collected. The endogenous cytokinin and gibberellin levels were measured as described in Kojima et al.^30^.

### GA20-oxidase activity tests

The coding sequence of each GA20ox gene was cloned into the *Eco*RI and *Xho*I sites of a modified pGEX-6p-1 (Novagen) vector. *E. coli* Rosetta (DE3) pLysS (Novagen) cells were used for protein expression. The bacterial culture (5 mL) was grown at 37 °C overnight and inoculated into fresh LB medium (500 mL) containing 50 μg/mL ampicillin. After the bacterial culture reached an OD600 of 0.6–0.7 at 37 °C, 0.1 mM IPTG was added and the culture was incubated at 18 °C for 16–18 h. The cells were collected by centrifugation and sonicated in lysis buffer (10 mM Na-phosphate [pH 7.5], 150 mM NaCl, 1 mM DTT, Protease Inhibitor Cocktail [Roche]). After centrifugation, the supernatant from the resulting lysate was purified using glutathione Sepharose 4B (GE Healthcare). The resulting proteins were used for the enzyme activity tests. After enzymatic cleavage of the GST tag with PreScission Protease (GE Healthcare), the de-tagged proteins were purified on a Superdex 200 16/60 column (GE Healthcare) or further purified by passage through Glutathione Sepharose 4B resin to remove GST tag contaminants. Protein fractions were concentrated with an Amicon Ultra-4 concentrator unit (10 kDa molecular weight cutoff) and used for subsequent enzyme assays. The purified proteins were quickly frozen in liquid N_2_ and stored at −80 °C until use. In vitro enzyme activity assays were performed as described for rice GA20ox^31^. GA_19_ and GA_20_ were included in the reaction mixture as a substrate for GA20ox, respectively. Deuterated [17,17-^2^ H_2_] GA_4_ were purchased from OlChemIm and used as internal standards for the GA20ox reactions. Full-scan GC-MS analysis was carried out with a JEOL JMS- K9 mass spectrometer and Agilent Technology 6890N gas chromatograph.

### RNA sequence (RNA-seq) and data analysis

RNA was extracted from 2-mm stage young panicles of Koshihikari, NIL-*Prl5*^ST-1^, NIL-*Pbl6*^ST-1^, and NIL-*Prl5*^ST-1^ + *Pbl6*^ST-1^ and used. RNA-seq was performed as described by Minami et al.^32^. Statistical analysis was performed by R, using a *P*-value of 0.05 as minimum. The edgeR package was used to normalize raw data counts and to generate cpm values^33^. GO-term enrichment analysis was performed using GOseq package in R statistical environment as described by Nozue et al.^34^, using a *P*-value of 0.05 as minimum for statistically significant enrichment. All data generated and analyzed during RNA-seq analysis are included in Supplementary Data 1–3.

### Statistic and reproducibility

All the experiments were conducted in at least three biological replicates to ensure the reproducibility. Exact sample number was indicated in the figure legends. Mean from at least three independent biological experiments was presented in each figure, in which error bars represented standard deviation. A number *n* suggested biological replications indicated in the figure legends. Statical analysis was performed by using R (https://www.r-project.org/). Statistical differences were determined by Student’s *t*-test and one-way ANOVA with multi-comparison Tukey’s HSD post hoc test. A value of *P* < 0.05 was considered to be statistically significant. Significant differences are indicated in figures using a star. All experiments were repeated at least three time.

### Reporting summary

Further information on research design is available in the Nature Research Reporting Summary linked to this article.

## Supplementary information

Description of Additional Supplementary Files

Supplementary Information

Supplemantary Data 1

Supplemantary Data 2

Supplemantary Data 3

Reporting Summary

## Data Availability

The accession code for the raw sequencing data is GSE151043. The normalized data of RNA-seq are provided as Supplementary Data 1. The analyzed data of RNA-seq are provided as Supplementary Data 2 and 3. Newly generated plasmid is available from the corresponding author on reasonable request. The authors declare that all data supporting the findings of this study are available within the article, its Supplementary Information files and from the corresponding author on reasonable request.

## Code availability

Data analysis of RNA-seq was performed by R as explained in the Methods section. The code that supports the findings of this study is available upon reasonable request to the corresponding author.
