# Supplementary material for: Diverse panicle architecture results from various combinations of Prl5/GA20ox4 and Pbl6/APO1 alleles
Source: Commun Biol. 2020 Jun 11;3:302. doi: 10.1038/s42003-020-1036-8 (PMC7289860; doi:10.1038/s42003-020-1036-8)
Supplement: Supplementary file 1 — Description of Additional Supplementary Files [file 42003_2020_1036_MOESM1_ESM.pdf]

Description of Additional Supplementary Files

The data of RNA-seq are provided as Supplementary Data 1-3.

File Name: Supplementary Data 1
Description: Normalized data of RNA-seq

File Name: Supplementary Data 2
Description: Analyzed data of RNA-seq

File Name: Supplementary Data 3
Description: Results of Gene Ontology (GO) representation analysis
