# Supplementary material for: Diverse panicle architecture results from various combinations of Prl5/GA20ox4 and Pbl6/APO1 alleles
Source: Commun Biol. 2020 Jun 11;3:302. doi: 10.1038/s42003-020-1036-8 (PMC7289860; doi:10.1038/s42003-020-1036-8)
Supplement: Supplementary file 2 — Supplementary Information [file 42003_2020_1036_MOESM2_ESM.pdf]

Supplementary Figures

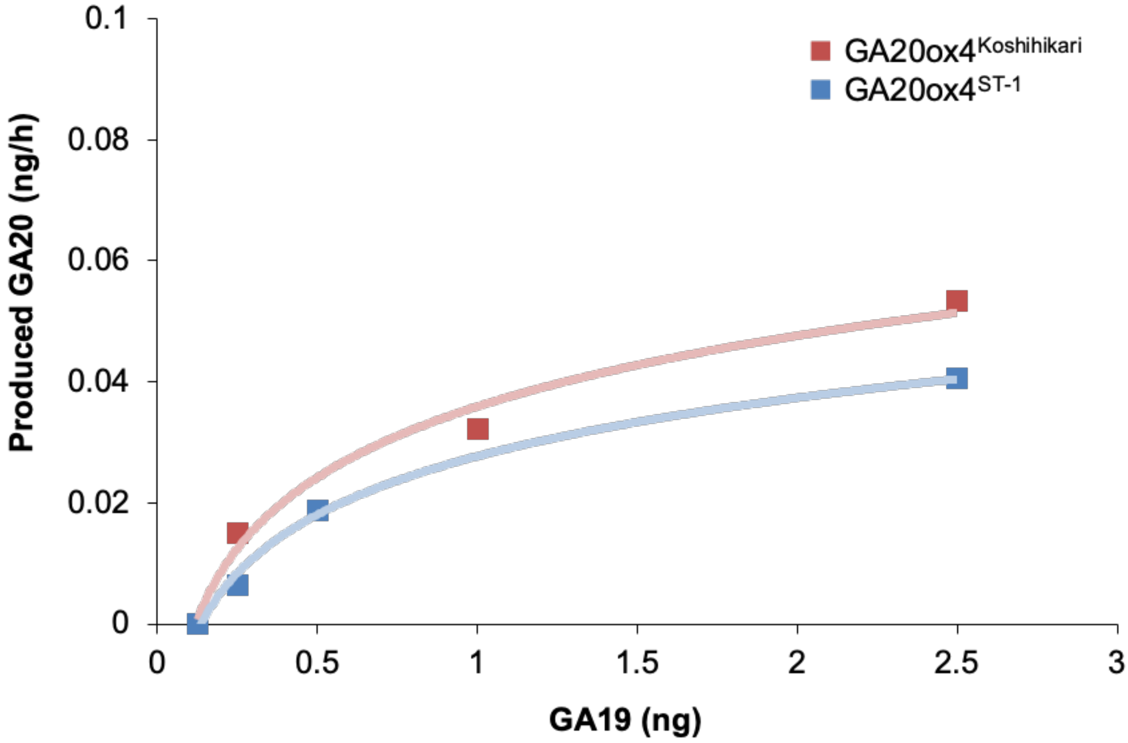

Supplementary Figure 1. Enzymatic activities of GA20ox4 proteins.

Enzymatic activities of recombinant GA20ox4^{Koshihikari} and GA20ox4$^{ST\text{-}1}$ proteins in 60-min reactions against GA19.

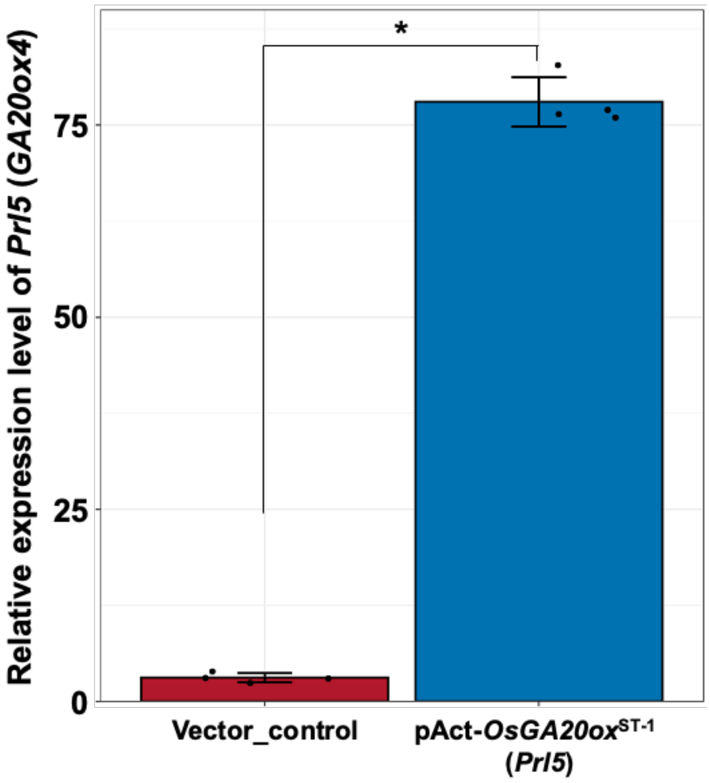

Supplementary Figure 2. Expression analysis using transgenic plants.

Expression analysis of *Prl5* (*GA20ox4*) using plants transformed with an empty vector (vector control) and expression vector for *OsGA20ox4*$^{ST\text{-}1}$ under the control of the *Actin* promoter. Relative expression levels were calibrated based on *Ubiquitin* expression. Error bars represent means ± SD (n = 4). * Significant at the 5% level (Student's t-test).

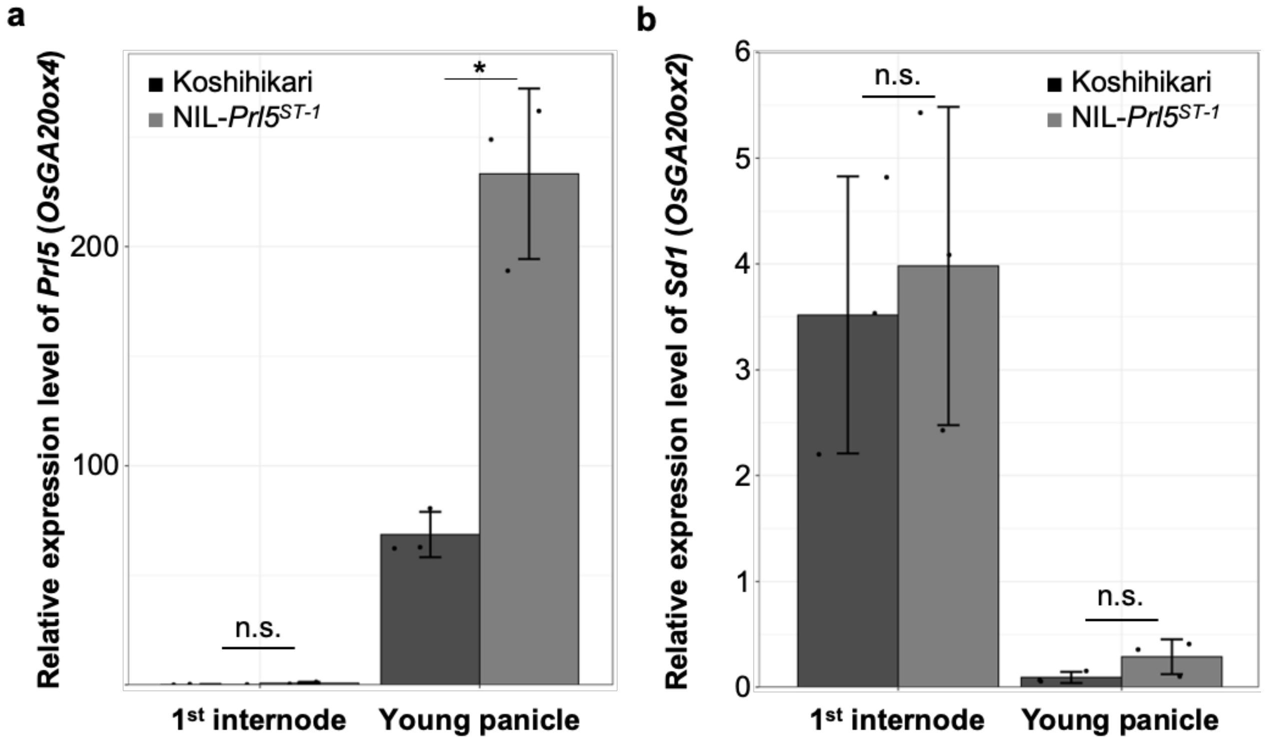

Supplementary Figure 3. Comparison of the expression levels between Koshihikari and NIL-*Prl5*$^{ST-1}$. Relative expression levels of *Prl5* (*GA20ox4*) (**a**) and *Sd1* (*GA20ox2*) (**b**) in first internodes and young panicles at the later stage of secondary branches differentiation. Error bars represent means ± SD (n = 3). * Significant at the 5% level (Student's t-test).

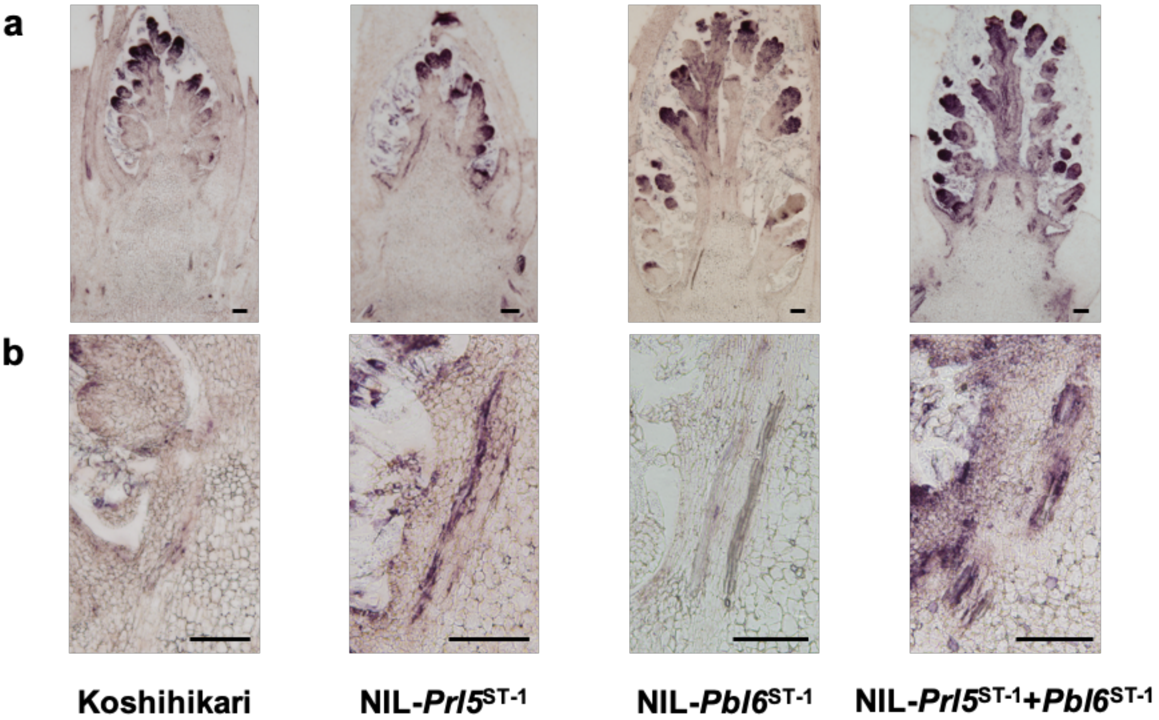

Supplementary Figure 4. Comparison of the expression patterns between NILs.
a-b In situ hybridization of *Prl5* at the later stage of secondary branch differentiation in Koshihikari and NILs. **b** shows close-up views of each vascular bundles. Scale bars: 100 µm.

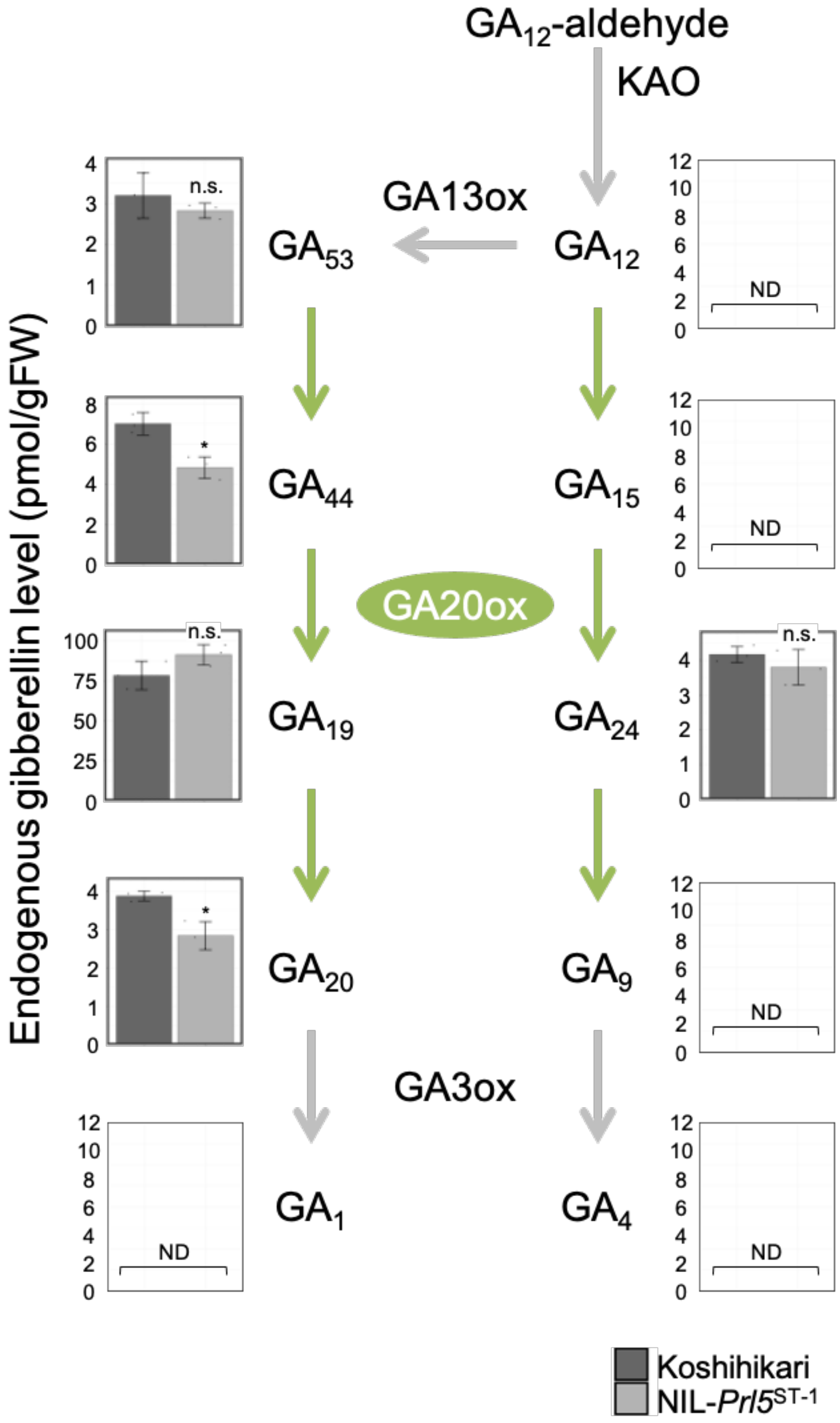

Supplementary Figure 5. Endogenous gibberellin levels in young panicles.

Comparison of the contents of GA biosynthesis intermediates in the early-13-hydroxylation pathway using 2-mm stage young panicles of Koshihikari and NIL-*Prl5*$^{ST\text{-}1}$. Error bars represent means ± SD (n = 3). ND: not detected. * Significant at the 5% level (Student's t-test).

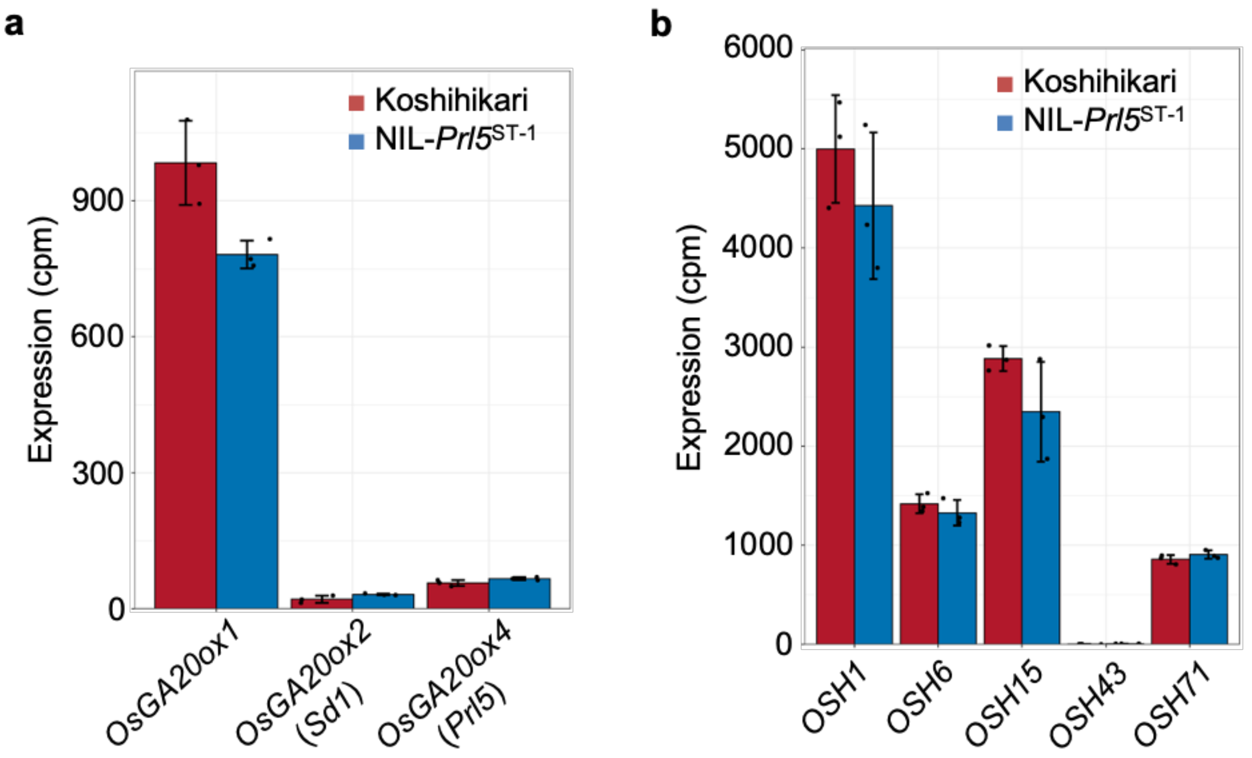

Supplementary Figure 6. Expression of *OsGA20ox* genes and *KNOX* genes in young panicles.

Expression levels of *OsGA20ox* genes (**a**) and *KNOX* genes (**b**) at 2-mm stage young panicles of Koshihikari and NIL-*Prl5*$^{\mathrm{ST\text{-}1}}$. Error bars represent means ± SD (n = 3).

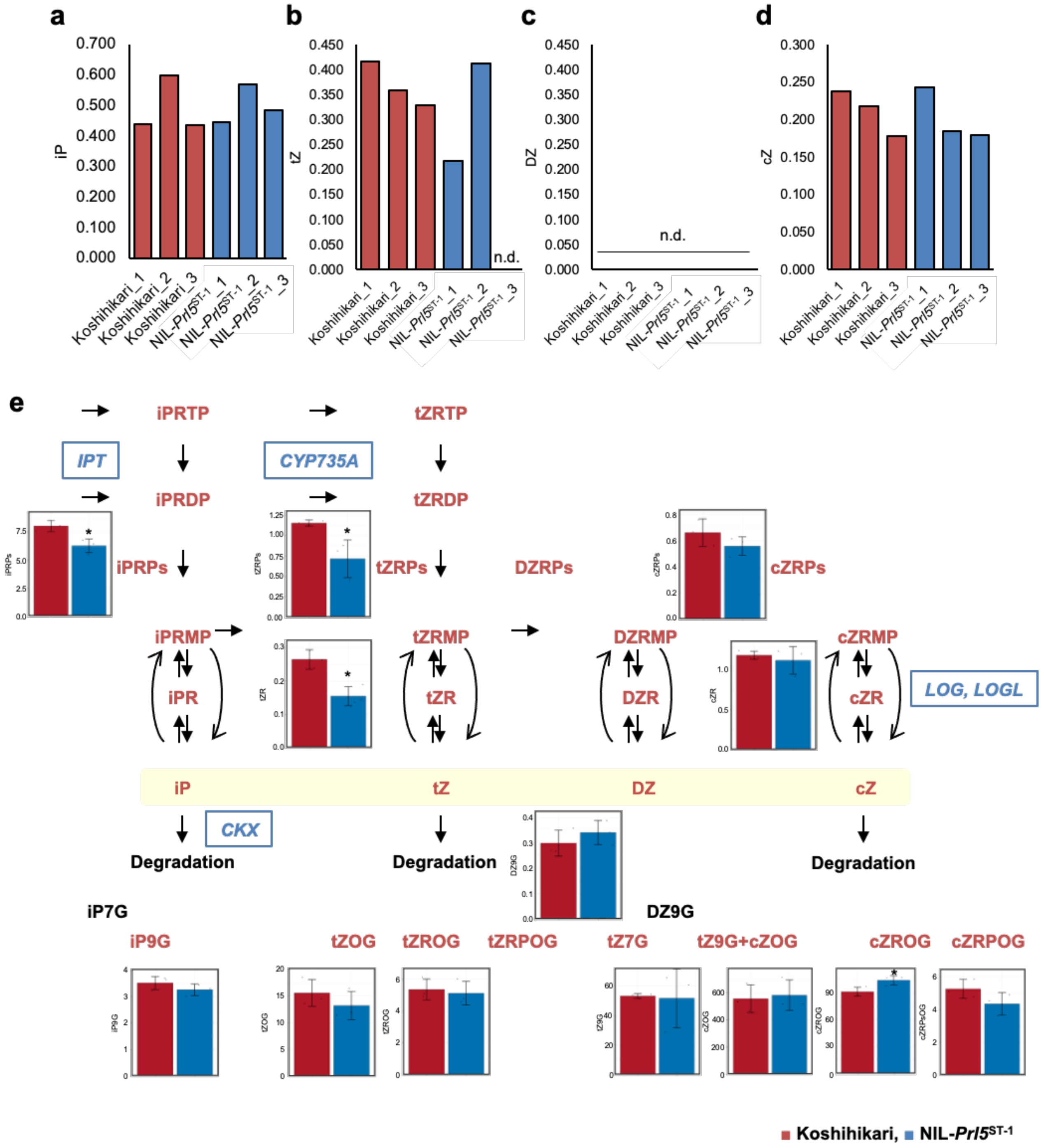

Supplementary Figure 7. Endogenous cytokinin levels in young panicles.

Comparison of endogenous cytokinin levels in 2-mm stage young panicles of Koshihikari and NIL-*Prl5* ST-1. **a-d** Active cytokinin, **e** Cytokinin biosynthesis and metabolism pathway. Error bars represent means ± SD (n = 3). ND: not detected. *Significant at the 5% level (Student's t-test).

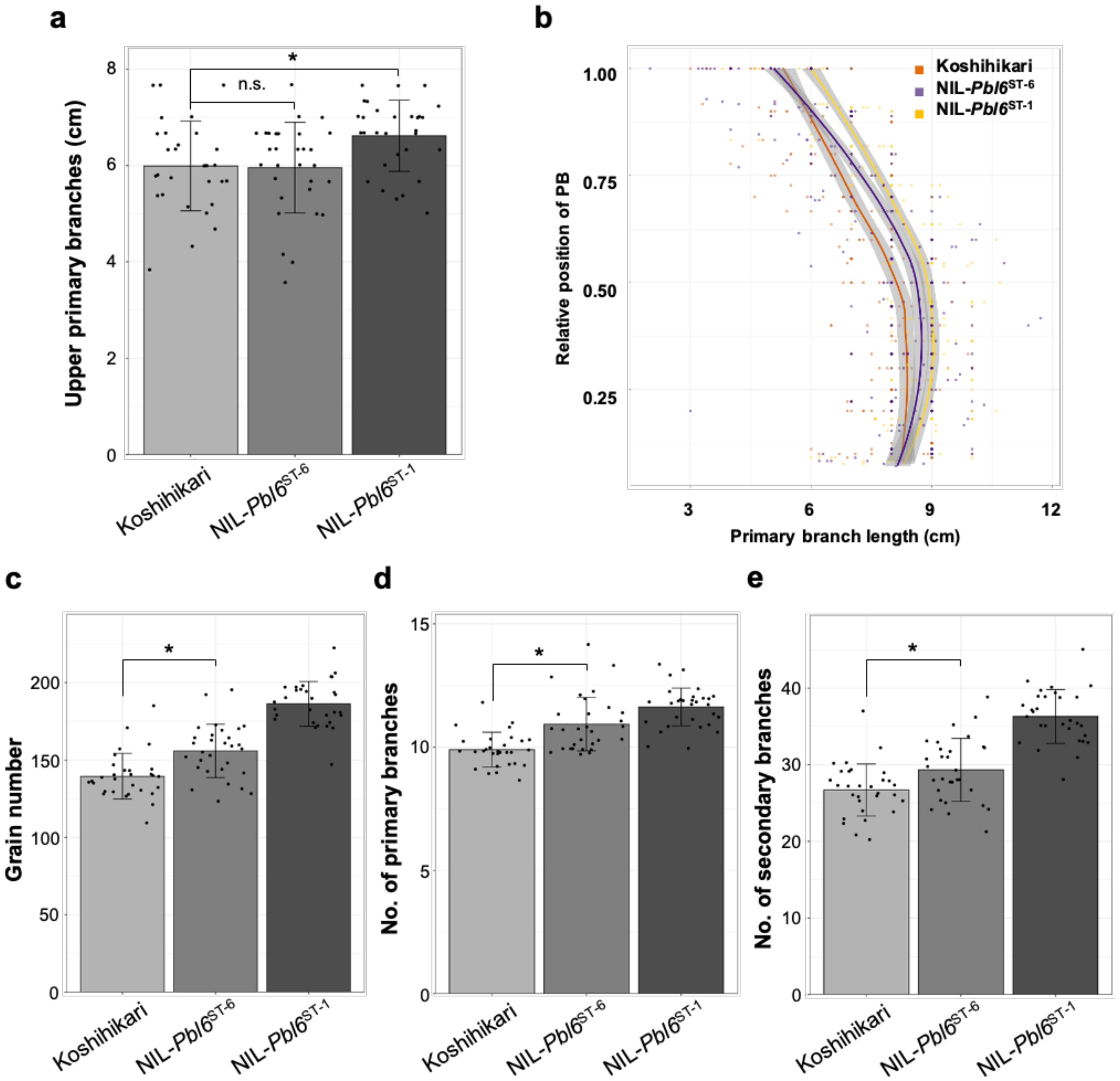

Supplementary Figure 8. Effects of *Pbl6*$^{\text{ST-6}}$ on panicle architecture under field conditions.

a Comparison of Lengths of the three upper primary branches. **b** Comparison of every primary branch length. Solid lines show regression curves. Orange line and dots indicate Koshihikari. Yellow line and dots indicate NIL-*Pbl6*$^{\text{ST-1}}$. Purple line and dots indicate NIL-*Pbl6*$^{\text{ST-6}}$. **c-e** Comparison of panicle traits. **c** Grain number. **d** No. of primary branches. **e** No. of secondary branches. n = 30 plants in A-E. Error bars represent means ± SD in **a**, **c-e**. * Significant at the 5% level (Tukey's significant difference test) in **a**, **c-e**.

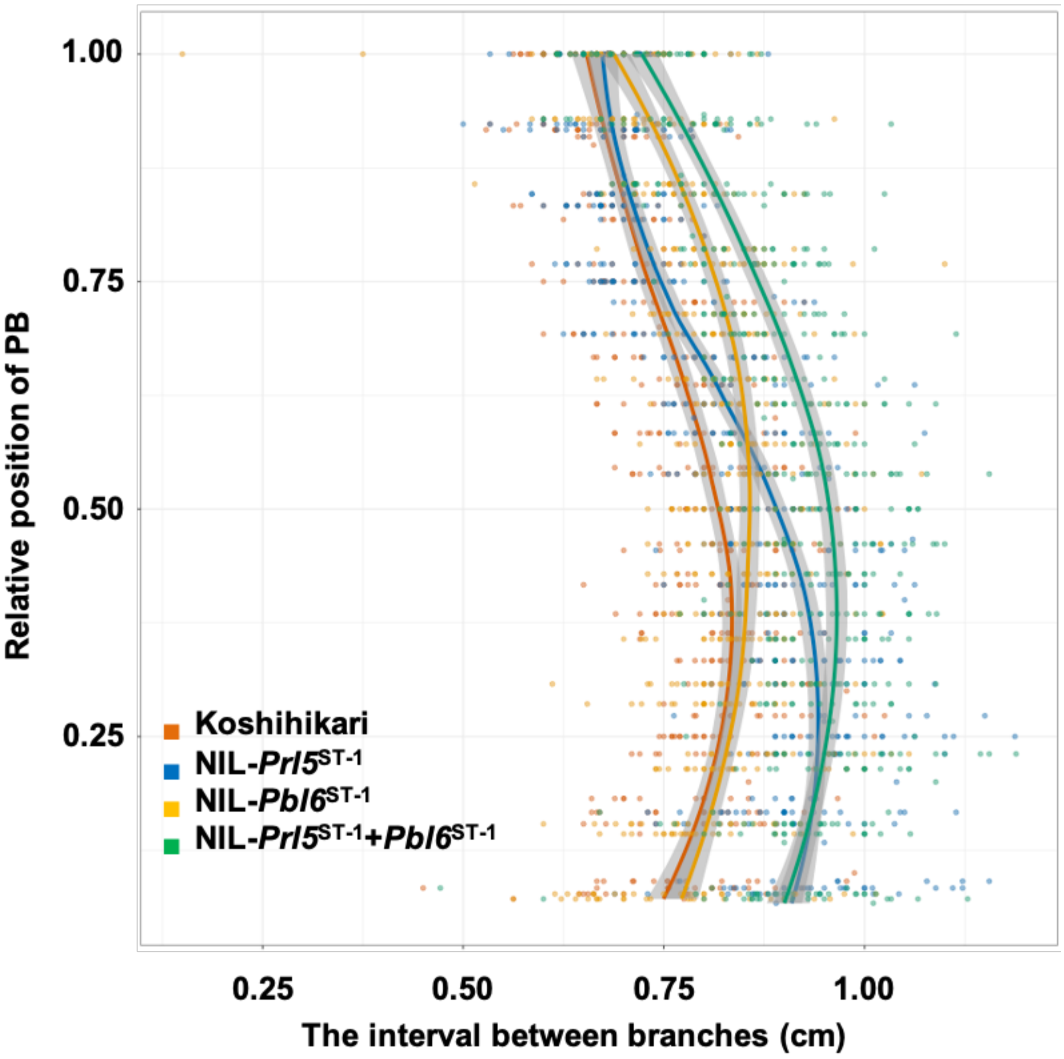

Supplementary Figure 9. Comparison of the branch density on each primary branch between NILs. Comparison of the intervals between branches of each primary branch. The value on the horizontal axis was obtained by dividing the primary branch length by the total number of secondary branch and secondary rachilla on its primary branch. Solid lines show regression curves. Orange line and dots indicate Koshihikari. Blue line and dots indicate NIL-*Prl5* $^{ST-1}$. Yellow line and dots indicate NIL-*Pbl6* $^{ST-1}$. Green line and dots indicate NIL-*Prl5* $^{ST-1}$ +*Pbl6* $^{ST-1}$. n = 40 plants.

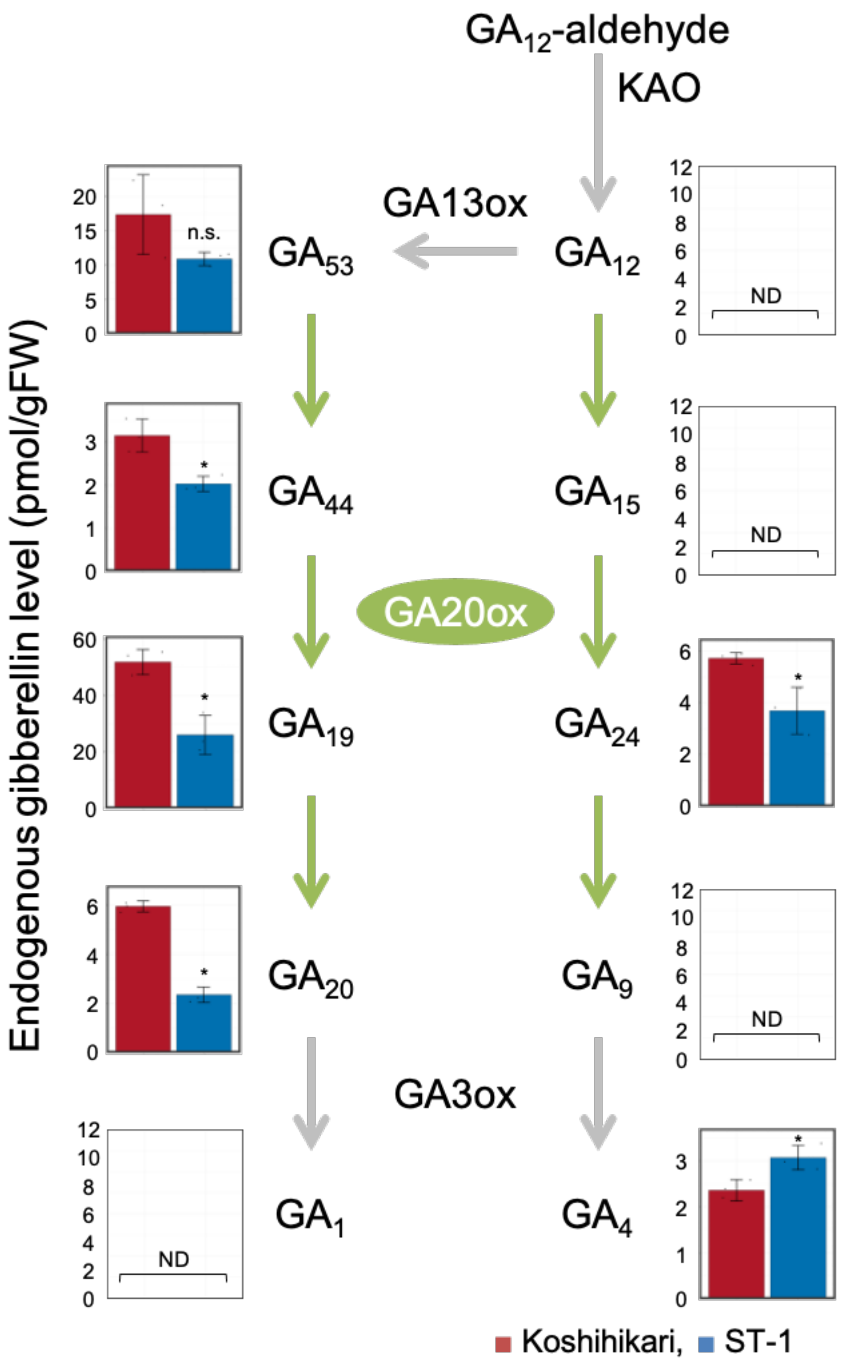

Supplementary Figure 10. Endogenous gibberellin levels in young panicles of Koshihikari and ST-1. Comparison of the contents of GA biosynthesis intermediates in the early-13-hydroxylation pathway using young panicles of Koshihikari and ST-1. Error bars represent means ± SD (n = 3). ND: not detected. * Significant at the 5% level (Student's t-test).

Supplementary Tables

Supplementary Table 1. Oligo sequences used for QTL analysis

Name	Chromosome	Forward Primer	Reverse Primer
1. QTL analysis			
RM3252	1	ATGCAAGCATCTGCTTATGG	GTTGGTAACTTTGTTCCCATGC
RM6324	1	CTGTACAAGAACGGCAGCAACC	GCACCACCAAACAGAGACAGAGG
Q1046	1	ATGTCAGACTCCGATCTGGG	TCTTGACCTTACCACCAGGC
Q1052	1	TTACCTCAGGCTCTTCAGGC	AGAAGTGAGCAATCATGGCC
RM259	1	GAAGTGCTCCCTAAACTTGTTGC	TTATGGAGGATGGATTCGAAGG
RM3627	1	CTATGCCTCCGCCTCTATCTCC	CTCTCTGGACCTACCCGTCATCC
Q406	1	GGGTCGGATAGCCACACAC	GAGGTAATCTCGCGGAGTTG
RM6696	1	CACTTGACGAAGATGAAGGATGG	GAGTGAACTCCTTTGATGGTTTGC
RM5310	1	GGGACCAAGACCTTTCCAATGC	GCGGAAGCAGGAGAATCGTAGC
Q1218	2	AGACATGCCAATGTGATGGC	TCGGTAGTATGGGGCTTGTC
Q1235	2	AACCATGGACAGTTGAACAC	CTCCGACAAGAACTTCCTC
Q1245	2	GGCATCTTCCCCTCTCTCTC	CCAACCCAAACCAGTCTACC
RM6639	2	ACCGGAAGGGATACTTATCAGC	CTTCCTGTGAAATAGTAGAGGTAGGC
RM5812	2	CGCTGACATCTTGCCCTC	GTAGGACCCACGTGTCATCC
RM341	2	CAAGAAACCTCAATCCGAGC	CTCCTCCCGATCCCAATC
RM5427	2	TGCTGTTGACACTTGACAGGTAGC	CACAATTATTGCGGCTCATCG
RM5651	2	CATGCTGATGCGATTAAGACTGC	CTAACCTGTGCCTTGTTTGATGG
RM5470	2	CGTGTATTGCATGGATTGTCTGG	CGGAACCCACAATTTCTTTCTGC
RM5631	2	CGTCCAAGAAATATTGCAGT	GTGAGACAGAATCCTTACGC
RM207	2	ATCCTAGTGGATAAGGCACAGACTGG	CCCTTGCTCTTCCACCTCATCC
RM3372	3	CAAAGAATCCAAGGAGGCCAAAGC	ATGCCGATGAGCACCCAGAGG
RM6829	3	CGATGAAGAGCCAATCCTTCAGC	TGCTCGTCCCTTCTACAAACAGG
RM3126	3	CCTCCTTCGTCTTCTCCTTTGC	CTCCGGTACGTCGAAACTCG
RM5442	3	AAAGCCTTCCATCCAACCATCG	CAGTAGTGTTGCTGCAGTGAGGTAGG
RM5444	3	AGTCGCTGGTTCGCTTCATCG	ACTCACTGCACCCGTGATTTCC
RM1022	3	GTCTTTGATAGCGGCTTTGTCC	GGATGAGGGAGTAATGTCTCTTGG
Q1392	3	GTTGAACATATTGTGGGGGC	GGTTCTGGACTACATCGAACG
RM8208	3	GCCCAAACTACACTCTCTTG	GTAAATGCCTGAGTGCCTAC
RM347	3	CACCTCAAACTTTTAACCGCAC	TCCGGCAAGGGATACGGCGG
RM15770	3	AAGTGAGGCGACGAGGACGAAGG	AAACGCAACGCACAGAAGGAAGG
RM1350	3	AGGAACACCCAAGAGAGTCATGC	GCAAGAAAGCTCTGCTCCATGC
RM6987	3	ACGACACTCGTAGGTTCGTTCTTGC	TGCAGCATCGCAGCAATATCC
RM5688	4	GGTGATGATGAGTGTTTGATGC	TGACAGTAGTAGTTCAGCAGTGTCC
Q1516	4	TTGGTTGCTTCCTCCCATAC	GGCATTGTACGACGGATCTC
Q300	4	AAGTACTCTCCCGTTTCAAA	CCTCCCATAAAAATCTTGTC
RM1248	5	CTCTCAGGTGTGTTGTACATTGTTCC	CTGCTCAAACAAGCAGCTAATGG
RM169	5	CACCTCCTCCAAGATCCTTATGC	CTCTCTGTCTCGCTGTCTGTTGC
RM1115	5	CCACCACCATCTATCTGCATCC	CGTACAGTCAGTGAGCAAGACAGG
RM1237	5	CAGCACACATACTCTGGCTCTCC	CCGCGAGCTTTAGAAGAGAAGG
Q1568	5	GGCCCACATGTCAGTTACAC	CCCACCAGCCTCACTTACTG
RM3348	5	CTTCTCGGTTCATCCAAAGAGC	GTGGAAGCTATGGGTAGCTCACG
Q1608	6	TACTACCACTTCTAGATGAGTTCAG	CTGAATACACTTCAATTTCTCTC
Q1619	6	TCTCGAACTCTCCATCTCGG	CGAAGGAGGGGAGGTAAGAG
RM7193	6	ATGTGGGAATTTCTAGCCCC	CCCTAGTTTTCCAAATGGCC
RM3827	6	TAGGTAGGACCGTGCTTCATTGC	CCCTGGCCTTTCTTCAATCTGC
RM5753	6	GCACCATCCGTCAGAAACAGC	TCCGAGAAAGAAGAACCGCTAGG
RM3325	7	GGAGAAAGGAAGAGAGTGCATTGG	TTCCAAACACCCAGAGACAAAGC
RM5752	7	TTGCAATTAATTCGATCTCC	GCAGATCGATTCGTTAGTTC
Q1709	7	TTTCGGGAGGGATTATGACC	GTGACCCCCGATACAAACAC
RM7121	7	TACCAGCTGCATGTTACCCGATACC	CGGAATCAAATTCCAGCAACAGC
RM3404	7	CTCCTCAGTCCTGAGTCTCCTGTCC	CCCAGAGAGATTACACAGAGCAAGC
RM5847	7	CTTTAGGTAGCGTCATCTTCC	TGGAAATACAGAAGGAGTCG
RM5647	8	GTTCCATCCAGACATTGTAGAAGC	TAAACTTGGTCGTGGACAGTGC
RM8271	8	AGCAGCTCCGATTGTGTTAGCC	AATGGCGTCTGTGGTACTTTGC
RM3153	8	GTGTGATGGTGACGGATTACATGTGC	CATGCTGCAGAATTTCCATGTTGG
RM7049	8	GTGGATCAAACGCAGCTAATAACC	TGAGTTGAGCAAACGTCTGTTGG
RM6635	8	CAAATACTTCTCCATCTGACCGTACC	GTTGCTGTCGATTCGGATTAGG
RM6966	8	ACCAACAAATGGGTCGTATTGG	CTACGGATTTACCCAGAAACATGG
Q1869	8	AATCCACATTTGGCTTCTC	CGTGTAACTGTTTCGCTTG

Name	Chromosome	Forward Primer	Reverse Primer
RM5777	9	CGTCGCCATCTAGTTCTCTCACC	CGAGAGTGCATGTGGAGTGAGG
RM5652	9	GTCCTGAGTGACTCGATAGCG	CACGCGTACGTAGCAGAGAG
RM7289	9	GGCCCACGACTTAATAGACATCG	ACAGCGAACGTGGTGTCTCC
RM3823	9	CTCCTTCAGTCGGTCGTC	AAGGAGTCTGTCGCTTTACC
Q1907	10	AGATGGTTGCCAAGAGCATG	GTCACGTGGCGATTTAGGAG
RM5304	10	CATCTTGAATCCTCCTTCGACTCC	GGCAGCGATAGCAGGAAGAGG
RM6474	10	CGGCAAGAAGGTGAGCAGTGG	CGTTCTCATGGCGGAGCTTGC
RM1761	11	ATCTTACACTGTGCAGGTTGTGC	GGAACTTCCTTGGTGAATGAGC
RM3168	11	GGAGAAGAGAAGCTAGGGTTTAGGC	ATTAACGGCCCACGTATCATCG
RM7248	11	CAATGTCGCATGTGTATGTGTGG	GATGGAGGAAACAAGTCAACAACC
RM3701	11	GAAAGAGGAGGAAGAGCTAGAGG	CCATATGTACGGAGTGTGTTTACC
RM3428	11	GCCATTGACACCAAATGATCACC	GGCATATAAGGTCCATGGTGAATTGG
RM5582	11	AGGCGTGTGTGTGTCTTTTG	GAAACTCTGTACCTCCCTTGTG
RM6534	11	GGAGGCGTCATCATAGTCATGG	AAACTCCCAAACTCCTCCATTCC
RM6094	11	CGATTGCGATGGCGATTAGG	GAATCGGTGGAAGAGGTGACG
RM1302	12	GAACGTGAAAGAGAAGCTCATGG	TCTCCTTCTCTCCCAACATCTCG
RM247	12	AAGGCGAACTGTCCTAGTGAAGC	CAGGATGTTCTTGCCAAGTTGC
RM7344	12	CGACGCCATATATGCCTTTCTTCC	GCAAAGCTCTGACTGCCTCAGC
RM6945	12	TATACGGCAGACACACTGCATCC	CGAATCAGGTCCAGATCAGTCG
2. Fine mapping			
RM430	5	GTCCCTGATCAGAAACGAGATGG	TAGGGTTGGAAGAATGCAAGACC
RM18661	5	GTTCTTCTCCCTCCTCCTCTGC	ACACGCTACTGAAAGCTCATTGC
RM18704	5	GAGTGATGGCATTGCTTGAGAGC	CGAGCCATCATACCTCCGTCTAGC
RM18711	5	ACCTGCTCACCACAATTTGATTCC	TTCGAGCAATCTAGCCTGAGAGAGG
RM18713	5	GGAGGTAGAGGATGACGATCTGG	TTATTGTGGGAAGGCAGGAAGC
RM18717	5	CAGCCTTGGTAGCTGGATAATCG	CGTCTACTGCTCGAGAGAACTGTGG
RM20469	6	ACTTGATTGCAGCCTGGTCATAAGC	ACAGGCTGCTGCAAGGTAAAGC
RM20473	6	GAGGAAGGGAAGGAGAGGAAGG	CTGATCGGTTAAGTCCCAGACC
RM20487	6	GAAGCTTGCACATGTGGATTACC	CTTTGCAGCTGATTTGGATTGG
RM20512	6	GCGTCGGACTTACGTGAGAAGG	CTCAGCATGGAGACGAGAGACG
RM3430	6	AGCGAGAGCCACCTAATCTTGG	CTGCTCTTCTCCACCACGAACC
qPBL6_3	6	GCAACGCGAGAATATATACAGTG	ACGAAGGGAACTAGGGAAGC
qPBL6_4	6	ccaagtactccctccgtttc	caatgtccggtgtctctagc
qPBL6_6	6	CTCGTAGCGTACGAGGCTG	CATATGAATGCGTATGTATTGCTG
qPBL6_7	6	CAGTCAAACGTTGAACACGAA	TGGCCACTTGTCATAGTGCT
qPBL6_1	6	GAATCACATATGATAGAGCGAGCTT	TATTATCCGATCCGTTTTCACC
RM5509	6	CCTGTCAATTGATGATCCATGC	TACCTTCCAGCAGAAAGAAGACG
RM3138	6	TTGACAAGAGATCAAGGCGG	GTGAATGTTGAGCTGCATGG

Supplementary Table 2. Oligo sequences used for sequence analysis

Name	Sequence 5'-3'
Prl5_PRO_seq_1F	CGCTGCTGAACCACAACCGCCT
Prl5_PRO_seq_2F	GAAGGAGCCATGGGAGGGGAAG
Prl5_PRO_seq_3F	GCCTTAAACGTAACAATCAGAT
Prl5_PRO_seq_4F	CAATTACTCAACAAGAGGAAGT
Prl5_PRO_seq_5F	TGTGAGGATCCAGTATGACATC
Prl5_PRO_seq_6F	ACGGTCCTTAAGTACCTGGGTG
Prl5_PRO_seq_7F	TGCATCGCGAGTCCGTATCATC
Prl5_PRO_seq_8F	AAGTAGCCGATTCGAATTCGAG
Prl5_PRO_seq_9F	TATTTAGGCCTTGTTTAGTCAG
Prl5_Code_seq_1F	CCTTCTTGACAGTGAAAACCAGGC
Prl5_Code_seq_2F	GCTGGACGTGCCCGTGGTCGAC
Prl5_Code_seq_3F	TTCACAGAAATCACTAGTTAGA
Prl5_Code_seq_4F	AACCGGGCGATGACATGGAGCA

Supplementary Table 3. Oligo sequences used for RT-PCR

Name	Sequence 5'-3'
Prl5_F	GCCGACTTCATGCGCTTC
Prl5_R	ACTAGTATACATGGACAGTGAGC
Pbl6_F	GTCATCTGAGTTGGTAGTGTG
Pbl6_R	CAACAGATCTCATGGCAAG
Sd1_F	CGCCGAACGGAACGAAACGG
Sd1_R	GTAATAGAGAGAAGCCCAAC

Supplementary Table 4. Oligo sequences used for in situ hybridization

Name	Sequence 5'-3'
Prl5_in situ_F	ATCTCGAGCAGGTGAAGTCCGGGTAGAG
Prl5_in situ_R	ACGATATCAGGTGACGAAGGCGATAATG
Pbl6_in situ_F	ATCTCGAGCGACGAGCTCATGCAGTAGA
Pbl6_in situ_R	ACGATATCCTCCCCTTCTTCGCCTTC

Supplementary Note

Discussion on the accumulation of bioactive GAs in NIL-*Prl5*.

In the quantification of GA levels using Koshihikari and NIL-*Prl5*, the active GAs, GA1 and GA4, were not detected (Supplementary Fig. 5). Since *Prl5* isolated by QTL analysis showed moderate effects and NIL was used for this analysis, it is quite difficult to detect the difference of the accumulation.

Then, to confirm that the increased expression of *Prl5* in young panicles leads to the increased accumulation of bioactive GA, we compared the accumulation of GAs using parental lines, Koshihikari and ST-1. It was found that the accumulation of precursors decreased and that of GA4 increased in ST-1 (Supplementary Fig. 10). In addition, RNA-seq analysis revealed that introduction of *Prl5* altered the expression of GA related genes such as *EXPANSIN*. Based on these results, we concluded that the accumulation of bioactive GAs increased in NIL-*Prl5*.
